# Architecture and ssDNA interaction of the Timeless-Tipin-RPA complex

**DOI:** 10.1093/nar/gku960

**Published:** 2014-10-27

**Authors:** Justine Witosch, Eva Wolf, Naoko Mizuno

**Affiliations:** 1 Department of Structural Cell Biology, Max Planck Institute of Biochemistry, Am Klopferspitz 18, 82152 Martinsried, Germany; 2 Department of Physiological Chemistry and Center For Integrated Protein Science Munich (CIPSM), Butenandt Institute, Ludwig Maximilians University of Munich, Butenandtstrasse 5, 81377 Munich, Germany; 3 Institut für allgemeine Botanik, Johannes Gutenberg-University, Johannes-von-Müller-Weg 6, 55128 Mainz, Germany and Institute of Molecular Biology (IMB), Mainz, Germany

## Abstract

The Timeless-Tipin (Tim-Tipin) complex, also referred to as the fork protection complex, is involved in coordination of DNA replication. Tim-Tipin is suggested to be recruited to replication forks via Replication Protein A (RPA) but details of the interaction are unknown. Here, using cryo-EM and biochemical methods, we characterized complex formation of Tim-Tipin, RPA and single-stranded DNA (ssDNA). Tim-Tipin and RPA form a 258 kDa complex with a 1:1:1 stoichiometry. The cryo-EM 3D reconstruction revealed a globular architecture of the Tim-Tipin-RPA complex with a ring-like and a U-shaped domain covered by a RPA lid. Interestingly, RPA in the complex adopts a horse shoe-like shape resembling its conformation in the presence of long ssDNA (>30 nucleotides). Furthermore, the recruitment of the Tim-Tipin-RPA complex to ssDNA is modulated by the RPA conformation and requires RPA to be in the more compact 30 nt ssDNA binding mode. The dynamic formation and disruption of the Tim-Tipin-RPA-ssDNA complex implicates the RPA-based recruitment of Tim-Tipin to the replication fork.

## INTRODUCTION

DNA replication relies on the coordinated action of replisome components including a helicase, a primase, replicative polymerases and regulatory proteins (1). Regulatory components, such as the fork protection complex (FPC), ensure correct duplication of the genome (2). The FPC is thought to coordinate DNA unwinding and DNA synthesis by mechanically bridging and thus stabilizing the individual components within the replisome (3).

Timeless (Tim) and the Tim interacting protein (Tipin), which constitute the FPC, play a crucial role in DNA replication as an adaptor unit for several replisome proteins (4). Mammalian Tim (mTim) was originally thought as an ortholog of the circadian clock protein *Drosophila* Tim, but later it was identified as a FPC component involved in maintaining genome stability (5,6). Tim and Tipin are required for their mutual stabilization and nuclear localization (7). The importance of Tim-Tipin as a fidelity factor for DNA replication has been reported by several studies (3,8–12). Tim and Tipin were shown to interact with the MCM2–7 helicase as well as replicating DNA polymerases (3,10). The complex inhibits the helicase activity of the CMG (Cdc45-Mcm2–7-GINS) complex (9), but stimulates the activities of the DNA polymerases α, δ and ϵ (8,9). The depletion of Tim-Tipin causes uncoupling of polymerase-helicase, resulting in the accumulation of unwound single-strand DNA (ssDNA) covered by replication protein A (RPA) (11,12). These findings lead to the hypothesis that Tim-Tipin may physically stabilize the replisome by bridging the helicase and polymerase (4).

Tim-Tipin was also shown to interact with components of the DNA replication checkpoint, such as Chk1 and ATR-ATRIP (6,13–15). Knockdown of Tim and/or Tipin leads to reduced activation of ATR-Chk1-dependent signaling under replication stress conditions (6,13–15), slower DNA synthesis (3,14,16) and increased incidents of chromatid breaks, translocations and sister chromatid exchanges (12), supporting the essential role of the Tim-Tipin complex during DNA synthesis and checkpoint signaling.

Another important factor stabilizing the DNA replication fork is RPA. RPA covers and protects exposed ssDNA from nucleases and prevents it from forming secondary structures. RPA is composed of three tightly associated subunits referred to as RPA70, RPA32 and RPA14 (Supplementary Figure S1A). The DNA-binding domains (DBD) of RPA70 (DBD-A, -B, -C) and RPA32 (DBD-D) have been characterized biochemically and structurally (17,18). They form a stable complex with RPA14 as RPA's DNA-binding core (19,20). Three additional modules, namely, RPA70N (21,22), RPA32N (23,24) and the RPA32 winged helix (WH) domain (25), have been identified to interact with other binding partners, but these domains are not incorporated in the structural core (26,27).

RPA uses two discrete ssDNA binding modes with a footprint of 8 or 30 nt (28,29), which are recognized by the DBD-A and -B (8 nt mode) and all four DBDs A–D (30 nt mode), respectively (20,30). These two RPA binding modes differ in the affinity to ssDNA with dissociation constants (*K*_D_) of ∼50 nM for the 8 nt mode and ∼0.05 nM for the 30 nt binding mode with a cooperativity of *ω* = 10–20 (28,30,31), and they coexist in a dynamic equilibrium in solution (32,33). RPA undergoes a progressive compaction as the coverage of RPA by ssDNA progresses (19).

The recruitment of RPA to the replication fork is proposed to depend on unwinding by the helicase (34,35). In turn, RPA provides a binding platform for additional factors during DNA repair (35,36). These include Tim-Tipin (3,37), and the DNA repair factors XPA (xeroderma pigmentosum complementation group A protein) (38), UNG2 (uracyl DNA glycosylase-2) (39) and RAD52 (40), which are reported to bind to the WH domain of the RPA32 subunit. Tim-Tipin cooperate with RPA to assure the structural integrity of the replication fork, however, the nature of this interaction is elusive.

In this study we explore the interactions of the FPC proteins Tim-Tipin with RPA and ssDNA using electron microscopy (EM) and biochemical approaches. The cryo-EM 3D reconstruction of a reconstituted 1:1:1 Tim-Tipin-RPA complex revealed a globular architecture of the complex, identifying a U-shaped domain covered by a RPA lid. RPA employs a compact conformation within the complex, resembling the long-ssDNA binding conformation (19,20). Biochemical examination of the Tim-Tipin-RPA complex on ssDNA shows that complex formation is modulated by the binding mode/conformation of RPA on ssDNA. This finding suggests that a conformational switch of RPA controls the recruitment of Tim-Tipin, which might have implications on the effective organization of DNA replication and DNA repair.

## MATERIALS AND METHODS

### Cloning and protein expression

RPA and Tipin genes were purchased from Thermo Scientific (Epson, UK), the Tim gene was obtained as a gift from Dr. Achim Kramer, Charité Berlin, Germany. Genes were amplified by polymerase chain reaction and cloned into self-generated pEC series vectors designed for Ligase Independent Cloning (41). Recombinant full-length mouse Tipin (BC016211, amino acids (aa) 1–278) and mouse RPA32 (BC004578, aa 173–270 (RPA32WH)) were cloned as 3C protease cleavable hexahistidine (His) and Glutathione S-transferase (GST) fusion proteins.

Mouse Tim (AB019001, aa 1–1134), mouse RPA70 (BC019119, aa 190–623 (RPA70, DBD-ABC), aa 445–623 (RPA70C, DBD-C), aa 190–431 (RPA70AB, DBD-AB) and aa 1–623 (RPA70FL)), mouse RPA32 (BC004578, aa 43–270 and aa 1–270 (RPA32FL)) and mouse RPA14 (BC028489, full-length, aa 1–121) were cloned as 3C protease cleavable hexahistidin fusion proteins. The domain organization is depicted in Supplementary Figure S1A.

Protein expression was performed using Terrific Broth medium supplemented with appropriate antibiotics. *Escherichia coli* BL21 (DE3) gold (Stratagene, La Jolla, USA) was used for the co-expression of RPA70/32/14, RPA70FL/32FL/14, RPA70C/32/14 and RPA32/14. *E. coli* BL21 (DE3) *pLysS* (Stratagene) was used for the expression of RPA70AB, RPA32WH and Tipin. The co-expression of Tim-Tipin was performed using *E. coli* BL21 (DE3) gold containing the pRARE plasmid (Novagen, Darmstadt, Germany).

Bacterial cultures were grown at 37ºC until they reached an optical density of 1.8–2.2. The temperature was then reduced to 18ºC and protein expression was induced with 0.1 mM Isopropyl β-D-1-thiogalactopyranoside (IPTG) for 16–18 h. The expression of RPA70AB was induced with 1 mM IPTG for 4 h at 37ºC. The cells were harvested by centrifugation (8000 × *g*, 10 min) and kept at −80ºC until further use.

### Protein purification

The pellet of recombinantly co-expressed His-GST-Tipin and His-Tim was lysed in 50 mM Tris pH 7.4, 400 mM NaCl, 25 mM imidazole, 10% glycerol, 1 mM ß-mercaptoethanol (ß-ME) supplemented with 1 mM phenylmethanesulfonyl fluoride (PMSF) and 3 mg DNase I (Roche Diagnostics, Mannheim, Germany). The soluble fraction was loaded on Ni-NTA-sepharose, washed with buffer 1 (50 mM Tris pH 7.4, 200 mM NaCl, 25 mM imidazole, 5% glycerol, 1 mM ß-ME) and buffer 1 supplemented either with high salt (1.2 M NaCl) or adenosine triphosphate (2 mM adenosine triphosphate (ATP), 10 mM MgSO_4_, 50 mM KCl). The protein complex was eluted by an imidazole gradient to 500 mM and further purified using a Q-sepharose column (GE Healthcare, Freiburg, Germany). His-tag and GST-tag were cleaved with 3C protease (final concentration 0.006 mg/ml) during over night dialysis (50 mM Tris pH 7.3, 150 mM NaCl, 14 mM ß-ME) at 4°C followed by size exclusion chromatography (SEC) using a Superdex 200 16/60 column (GE Healthcare) pre-equilibrated with 20 mM Hepes pH 7.0, 125 mM NaCl, 2 mM DTT. For GST-pull-downs the GST-tag was left uncleaved.

Cell pellets for recombinant His-tagged RPA70/32/14, RPA70FL/32FL/14 or RPA70C/32/14 were lysed as described above. The clarified fraction was loaded on Ni-NTA-sepharose and the beads were washed with high salt and ATP prior to the elution. The eluted complex was desalted using a Sephadex G-25 Fine desalting column (GE Healthcare, Freiburg, Germany) to 25 mM Bis-Tris pH 7.0, 150 mM NaCl, 5% glycerol, 14 mM ß-ME, 0.01 mM Zinc acetate for RPA70/32/14, 25 mM Bis-Tris pH 7.3, 100 mM NaCl, 5% glycerol, 14 mM ß-ME, 0.01 mM Zinc acetate for RPA70FL/32FL/14 and 20 mM Tris pH 7.6, 100 mM NaCl, 5% glycerol, 5 mM DTT, 0.01 mM Zinc acetate for RPA70C/32/14, applied on a Q sepharose column (HiTrap Q HP, GE Healthcare) and washed with 190 mM (RPA70/32/14, RPA70FL/32FL/14) or 140 mM NaCl (RPA70C/32/14). The complex was eluted using a linear gradient up to 350 mM (RPA70/32/14, RPA70FL/32FL/14) or 300 mM NaCl (RPA70C/32/14). The proteins were further purified by SEC using 20 mM Hepes pH 7.0, 125 mM NaCl, 2 mM DTT, 0.01 mM Zinc acetate.

For the purification of RPA32/14 the clarified cell lysate was loaded on Ni-NTA-sepharose and desalted after elution into 25 mM Bis-Tris pH 7.0, 100 mM NaCl, 5% glycerol, 10 mM ß-ME. The protein was further applied to Q-sepharose column (HiTrap Q HP, GE Healthcare), washed with 200 mM NaCl and eluted with a linear gradient up to 1 M NaCl. The protein eluted between 350 and 450 mM NaCl as a single peak. The His-tag was removed by incubation with 3C protease over night at 4°C and further purified by SEC (20 mM Hepes pH 7.0, 150 mM NaCl, 5 mM DTT).

For the purification of RPA32WH the protein was bound to GSH sepharose (GE Healthcare). For the purification of RPA70AB the protein was captured by Ni-NTA. After elution the His-tag or GST-tag was cleaved with 3C protease and the protein was passed through a Q-sepharose column. The flow through after Q-column containing RPA32WH or RPA70AB was further purified by SEC (20 mM Hepes pH 7.0, 100 mM NaCl, 2 mM DTT).

The purity of the complexes was assessed by monitoring the absorbance at 280 and 260 nm during ion exchange chromatography and SEC, the 260/280 ratio and sodium dodecyl sulphate-polyacrylamide gel electrophoresis (SDS-PAGE).

### GST-pull-down assay

Note that 6 μg of GST-Tim-Tipin complex (4.2 μM final concentration) was mixed with 2-fold molar excess of RPA (input sample) in 30 μl, supplemented with 2× buffer to a total volume of 60 μl containing the final concentrations of 20 mM Hepes pH 7.5, 125 mM NaCl, 12.5% glycerol, 0.1% NP-40, 1 mM DTT, 0.01 mM Zinc acetate and incubated at 30°C for 20 min. Note that 12 μl GSH-sepharose beads (GE Healthcare, 50% v/v) and 200 μl buffer P1 (20 mM Hepes pH 7.5, 125 mM NaCl, 12.5% glycerol, 0.1% NP-40, 1 mM DTT, 0.01 mM Zinc acetate) were added and the proteins were immobilized for 1 h under constant rotation at 4°C. Beads were washed three times with buffer P1. The bound proteins were eluted using 20 μl buffer P2 (30 mM Tris pH 8.8, 150 mM NaCl, 14% glycerol, 0.1% NP-40, 1 mM DTT, 0.01 mM Zinc acetate, 2 mM imidazole, 20 mM Glutathione). The eluate was analyzed on 18% SDS-polyacrylamide gels and stained with Coomassie blue.

### Electrophoretic mobility shift assay (EMSA)

All DNA substrates were fluorescently labeled at the 5′-end with fluorescein (5-FAM) and purchased from Purimex (Grebenstein, Germany) or Eurofins MWG Operon (Ebersberg, Germany): 8 nt, 5′-FAM-ATCCCTAA-3′; 14 nt, 5′-FAM-GACGGCATCCCTAA-3′; 30 nt, 5′-FAM-ACGCTGCCGAATTCTACCAGTGCCTTGCTA-3′; 60 nt, 5′-FAM-ACGCTGCCGAATTCTACCAGTGCCTTGCT AGGACATCTTTGCCCACCTGCAGGTTCACCC-3′. For a typical EMSA reaction, 16 μM of protein was incubated with the indicated amounts of ssDNA (64, 32, 8, 4 μM ssDNA). Note that 7.5× buffer and 6× native loading dye (50% glycerol with bromphenol blue) was added to keep the final buffer concentrations at 20 mM Hepes pH 7.5, 125 mM NaCl, 2 mM DTT and 0.01 mM zinc acetate (Buffer A). After 15 min incubation at room temperature (RT) and 15 min at 4°C, samples were separated on native 4–12% Tris-Glycine gels in Tris-Glycine running buffer pH 8.3 (Invitrogen, Darmstadt, Germany). For the cross-linked conditions, RPA was treated with 2% (w/v) and Tim-Tipin and Tim-Tipin-RPA with 1% (w/v) glutaraldehyde for 20 min at RT. Before separation on native Tris-Glycine gels the reaction was stopped by adding Tris pH 7.5 to a final concentration of 80 mM. Gels were analyzed by fluorescence imaging (Typhoon FL 7000 phosphoimager, GE Healthcare, Freiburg, Germany) followed by Coomassie staining.

### Immunoblot

Samples were separated on native 4–12% Tris-Glycine gels in Tris-Glycine running buffer at pH 8.3 (Invitrogen, Darmstadt, Germany). The gels were incubated in 0.1% SDS for 15 min and the proteins were transferred onto 0.2 μm polyvinylidene fluoride (PVDF) membranes (Merck Millipore, Darmstadt, Germany) for 80 min at 200 mA. The transferred proteins were fixed to the membranes by incubation in 10% acetic acid for 15 min and air drying. After rehydration in 1× Tris-buffered saline (TBS) the membranes were treated with blocking solution (10% non-fat dry milk in 1× TBS) for 1 h at RT, incubated either with anti-RPA70 (sc-166023, Santa Cruz Biotechnology, Santa Cruz, CA, USA) or anti-Tipin (sc-160865, Santa Cruz Biotechnology) primary antibody for 1 h at RT and washed twice with TBS containing 0.05% Tween 20 (TBS-T) and once with TBS. Membranes were further incubated with horseradish peroxidase-conjugated anti-mouse or anti-goat secondary antibodies (Santa Cruz Biotechnology) for 1 h at RT and washed twice with TBS-T and once with TBS. Bound antibodies were detected by chemiluminescence using ECL reagents (GE Healthcare, Freiburg, Germany) and an ImageQuant LAS 4000 imager (GE Healthcare).

### DNA substrates for fluorescence anisotropy (FA)

FA was measured on a Genios Pro (Tecan, Männedorf, Switzerland) using 5′-fluorescein-labeled DNA substrates (see above) and purified Tim-Tipin complex. The final DNA concentration was 10 nM, while the protein concentration varied between 1 nM and 10 μM. The binding partners were incubated for 15 min at 30ºC in Buffer A in a total volume of 50 μl before anisotropy reading. The excitation and emission wavelengths were 485 and 535 nm, respectively. Each titration point was carried out three times using 10 reads with an integration time of 40 μs.

Data was analyzed with the Origin 8.1 software (OriginLab, Northampton, MA, USA). The *K*_D_ was calculated by directly fitting the curve using the Hill function (Equation (1)),

(1) \begin{equation*} A_{{\rm total}} = A_0 + (A_{\max } - A_0 )\frac{{x^n }}{{k^n + x^n }} \end{equation*}

where *A*_total_ is measured anisotropy, *A*_0_ is the intrinsic anisotropy of the DNA substrate, *A*_max_ is the anisotropy of the saturated protein-DNA complex, *n* is the Hill coefficient and *x* is the concentration of the protein. The quality of the regression was evaluated by agreement between the observed and calculated binding isotherms and residual plot analysis.

### Biochemical reconstitution of Tim-Tipin-RPA and Tim-Tipin-RPA with ssDNA

For the formation of the Tim-Tipin-RPA complexes Tim-Tipin was mixed with RPA at a 1:1 (16 μM:16 μM, ∼65 μg Tim-Tipin and 38.5 μg RPA) or 1:2 (16 μM:32 μM, ∼65 μg Tim-Tipin and 77 μg RPA) molar ratio in 25 μl final volume, incubated for 15 min at RT, for 15 min at 4ºC and loaded on a Superose 6 3.2/PC column (typical loading range 0.0005–0.5 mg) on ÄKTAmicro (GE Healthcare, Freiburg, Germany) equilibrated with Buffer A. For the reconstitution of Tim-Tipin and RPA32WH the molar amount of used protein mixture was increased by four (64 μM:64 μM, 64 μM:128 μM, Tim-Tipin:RPA32WH) because of low content of tyrosine or typtophane in RPA32WH and resulting low absorbance at 280 nm.

For the SEC analysis of RPA, Tim-Tipin and Tim-Tipin-RPA with ssDNA, the concentration of protein:ssDNA in 25 μl final volume was 16 μM:32 μM and 32 μM:8 μM for 60 nt ssDNA, 16 μM:32 μM and 16 μM:8 μM for 31 nt ssDNA and 14 nt ssDNA. The following oligonucleotides were used for SEC analysis on a Superose 6 3.2/PC column on ÄKTAmicro: 60 nt, CAGACCGCCACCGACTGCTTAGATATTTAAGTTTTCTAATTTTTCATTGAAAGCATTAAG; 31 nt, CGGGATCCCAGCCAGCGATGTCTCAAGCTGC; 14 nt, AGAAGAGCCAAAAC. The ratio of the absorption at 260 and 280 nm was calculated to estimate, if ssDNA was bound to Tim-Tipin, RPA or Tim-Tipin-RPA.

All SEC columns (Superdex 200 10/300 GL, Superose 6 3.2/PC, GE Healthcare, Freiburg, Germany) were calibrated using molecular mass standard proteins (Bio-Rad Laboratories, Hercules, CA, USA): Vitamin B_12_ (1.35 kDa), Myoglobin (17 kDa), Ovalbumin (44 kDa), γ-globulin (158 kDa) and Thyroglobulin (670 kDa).

### Static light scattering (SLS)

SLS of Tim-Tipin, RPA and Tim-Tipin-RPA was performed using a high pressure liquid chromatography system from Waters (Milform, MA, USA) (pump, ultraviolet (UV)) coupled to a TDA302 detector array (Viscotek, Malvern, Herrenberg, Germany). Note that 10 μL protein samples at a concentration of 2 mg/ml were injected into a Superdex 200 5/150 column coupled to the detector. Bovine serum albumin was used as a standard and the refractive index increment (dn/dc) was set to 0.180 ml/g for calculations. Data were analyzed using OmniSEC 4.5 software.

### Sucrose-gradient sedimentation

Sucrose gradients (5–20% w/v) were prepared in centrifuge tubes (Seton open-top polyclear centrifuge tubes, 14 × 95 mm) in 50 mM Hepes pH 7.5 and 150 mM NaCl using the Gradient Station (Biocomp, Fredericton, NB, Canada). Note that 200 μL of protein sample (1 mg/ml for Tim-Tipin or 3.5 mg/ml for standard proteins mixture) was applied on top of the gradient solution together with a 300 μl buffering cushion (50 mM Hepes pH 7.5, 150 mM NaCl, 2.5% sucrose). The sedimentation was carried out at 4ºC in a swing out rotor (Beckmann SW40 rotor) for 18 h at 35,000 revolutions per minute (rpm) (217,290 × *g*). Fractions were harvested using the Gradient Fractionator (Biocomp, Fredericton, NB, Canada) and analyzed by SDS-PAGE (4–12% Bis-Tris, Invitrogen, Darmstadt, Germany). Standard proteins (Bio-Rad Laboratories, Hercules, CA, USA) were used to calibrate the gradients: Myoglobin (17 kDa), Ovalbumin (44 kDa), γ-globulin (158 kDa for entire molecule of two light and two heavy chains) and Thyroglobulin (670 kDa for entire homo-dimeric molecule).

### Microscale thermophoresis (MST)

For MST measurements RPA was fluorescently labeled using the Cy3 protein labeling kit according to manufacturer's protocol (Jena Bioscience, Jena, Germany). The average number of lysines labeled per RPA complex was estimated to be 4.4. Note that 100 nM Cy3-RPA was titrated with varying amounts of non-labeled Tim-Tipin (25–80 550 nM) in Buffer A and incubated for 10 min at RT before measurements. The thermophoresis measurements were performed at 50% LED and 65% IR-Laser power using Monolith NT.115 (NanoTemper, München, Germany). Laser-On time was 40 s, and Laser-Off time was 5 s. The experiments were repeated three times.

For the data processing of the measurements the results were further analyzed using Origin 8.1 software (OriginLab, Northampton, MA, USA). The baseline was substracted from each individual experiment and each individual repetition was divided by its amplitude (the difference between the bound and unbound state). Three measurements were averaged and the standard deviation was determined. The *K*_D_ value was calculated by directly fitting the curve using the Hill function (Equation (1)) of Origin.

### GraFix and Mass spectrometry analysis (MS)

The cross-linking of protein complexes using glutaraldehyde (GraFix) was performed as described (42). Briefly, sucrose gradients (5–30% (w/v) for Tim-Tipin-RPA, 5–20% (w/v) for Tim-Tipin and RPA) combined with a glutaraldehyde gradient (0–0.2% (v/v)) were prepared in 50 mM Hepes pH 7.5, 150 mM NaCl using a standard gradient mixer and filled into centrifuge tubes (Beckmann, 50 Ultra-Clear Tubes, 14 × 95 mm).

Note that 200 μl of 1 mg/ml protein sample was applied onto the gradient together with a 300 μl buffering cushion (50 mM Hepes pH 7.5, 150 mM NaCl, 2.5% sucrose). The chemical fixation was carried out while sedimenting the sample at 12ºC in a swing out rotor (Beckmann SW40 rotor) for 16 h at 35,000 rpm (217,290 × *g*). Fractions were harvested from the bottom of the tube and the glutaraldehyde in the fractions was neutralized by adding Tris to a final concentration of 80 mM. Fractions of the cross-linked complexes were analyzed by SDS-PAGE (4–12% Bis-Tris, Invitrogen, Darmstadt, Germany).

MS analysis was performed to identify the presence of all components in the cross-linked samples. Each sample band was cut out, digested with trypsin (43), peptides were analyzed by Orbitrap mass spectrometry (44) and identified using Max Quant software (45). The stoichiometry of the protein complexes was determined by dividing the sum of all peptide peak intensities by the number of theoretically observable tryptic peptides (intensity-based absolute quantification (iBAQ)) (46).

For cryo-EM observation, sucrose was removed using a Zeba Spin Desalting Column (Thermo Scientific, Rockford, IL, USA) and samples were concentrated to 0.3 mg/ml using a Vivaspin 500 ultrafiltration device (Sartorius Stedim Biotech, Göttingen, Germany).

### Single-particle EM

For negative staining, samples (4 μl) were absorbed onto glow-discharged, carbon-coated copper grids for 30 s. Excess solution was blotted off with filter paper and the grid was washed four times with H_2_O before applying negative stain solution (1% (w/v) uranyl acetate). After blotting the grid was air-dried.

EM images were collected at 50 000× magnification with a defocus of 2–4 μm on a CM200-FEG (FEI, Eindhoven, The Netherlands) operated at 160 kV using an Eagle CCD camera (FEI) with a pixel size of 2.16 Å.

For cryo-EM, 5 μl of samples were applied to glow-discharged Quantifoil holey carbon grids, and vitrified in either liquid ethane or liquid ethane/propane mixture using a vitrobot cryo-station (FEI). The vitrified specimens were imaged using a Tecnai F20 electron microscope (FEI) operated at 200 kV and 50 000× magnification with a GATAN 626 cryo-holder. Data were collected with an Eagle CCD camera (FEI) with a pixel size at the specimen level of 2.21 Å.

### Image processing

Data processing was done using BSOFT (47), SPIDER (48), EMAN (49) and SPARX (50). For negative stained images, 13 311 particles for Tim-Tipin-RPA, 10 872 particles for Tim-Tipin, 1673 particles for RPA and 1914 particles for the non-cross-linked Tim-Tipin-RPA sample were selected, and for cryo-EM analysis 39 679 particles of cross-linked Tim-Tipin-RPA were chosen and these particles were boxed out with the size of 128 pixels. Contrast transfer function was corrected by flipping phases for the images of vitrified specimen.

For the initial assessment of the particles reference-free classification was performed from Tim-Tipin-RPA, Tim-Tipin and RPA using BSOFT or SPARX leading to 74 class averages. The alignment and classification was iteratively optimized.

Maps were displayed using UCSF Chimera (51). To estimate volumes of the subcomplexes, maps were segmented using the segment map option of the Segger package (52). To dock the crystal structures into the 3D map, the models were manually fitted using Chimera.

### Initial model generation/random conical tilt (RCT) processing

To obtain the first 3D map we used the RCT method by collecting pairs of tilted (45º) and untilted (0º) micrographs of negatively stained Tim-Tipin-RPA.

Out of 857 tilt pairs, 8 initial models were generated as described in Radermacher *et al.* (53). Two most similar models with prominent features were merged and used as the initial model.

Reprojections of the RCT reconstruction were compared to the class averages as well as raw data for verification. 3D reconstruction was improved using untilted 13 311 particles by iterative reprojection matching using merged RCT reconstruction as a reference.

### Cryo-EM reconstruction

Reference-based reconstructions were performed using SPARX. The reconstruction of the negatively stained images was low-pass filtered to 50 Å and used as initial reference. For the first cycles of iterative projection matching the data was binned by two (4.42 Å/pixel) and subjected to 3D reconstruction with 10 iterations per cycle and stepwise reduced angular increments from 5 to 2 degrees. For the last cycle of iterative refinement non-binned original data with 2.21 Å/pixel size was used for the final 3D reconstruction with an angular step of 2 degrees over the course of 10 iterations. For the estimation of the resolution the data set was splited into two and two independent reconstructions have been iteratively calculated using the RCT reconstructions as reference filtered to 50 Å. The Fourier shell correlation (FSC) curves between the two half-reconstructions were assessed (54). The final resolution was estimated to be 17.3 Å with FSC = 0.5 criteria. The amplitudes of the final density maps were corrected by using the amplitudes from an atomic model with a similar size/protein density (PDB code: 2BR2, exosome core) using the ‘bampweigh’ command in BSOFT and the structure was low-pass filtered to 17 Å. The angular distribution of the images used for the refinement was assessed using ‘sxplot_projs_distrib.py’ command in SPARX (Supplementary Figure S5D).

### Antibody labeling of the Tim-Tipin-RPA complex

Note that 0.23 μM of Tim-Tipin-RPA complex was mixed with 0.077 μM of antibody to RPA70 (sc-166023, Santa Cruz Biotechnology, Santa Cruz, CA, USA) and incubated for 30 min at RT. For Tipin localization 0.54 μM of Tim-Tipin-RPA complex and 1.25 μM of antibody to Tipin (sc-160865, Santa Cruz Biotechnology) were mixed, incubated for 60 min on ice and loaded on a Superose 6 3.2/PC column. Note that 4 μL of the mixture or the eluted immune complex was negatively stained with 1% (w/v) uranyl acetate. Images were collected as described above. Sixty-six particles for RPA70 and 347 particles for Tipin localization were boxed out with the size of 128 pixels. Image processing was performed as described above.

## RESULTS

### Tim-Tipin and RPA form a 1:1:1 complex *in vitro*

To analyze the interaction of Tim-Tipin and the RPA70/32/14 trimer quantitatively, we purified the individual components and reconstituted the complex (Supplementary Figure S1). Tim (residues 1–1134) and full-length Tipin (residues 1–278) were only stable when they were co-expressed and co-purified (Supplementary Figure S1B), in agreement with previous reports (9,14). SEC of the Tim-Tipin complex gives a single peak, showing a stoichiometry of 1:1 (Supplementary Figure S1C).

For our studies, we used RPA subunits RPA70 (DBD-A, -B, -C, residues 190–623), RPA32 (DBD-D and WH domain, residues 43–270) and RPA14 (residues 1–121). These constructs lack the RPA70 N-terminal domain (RPA70N) and the unstructured RPA32 N-terminus, which were shown to be structurally independent of RPA's DNA-binding core (19,22,25–27). The RPA DNA-binding core together with the WH domain (RPA or RPA70ABC/32D-WH/14) was co-expressed and co-purified as a trimeric complex giving a single peak in SEC (Supplementary Figure S1D).

**Figure 1.**
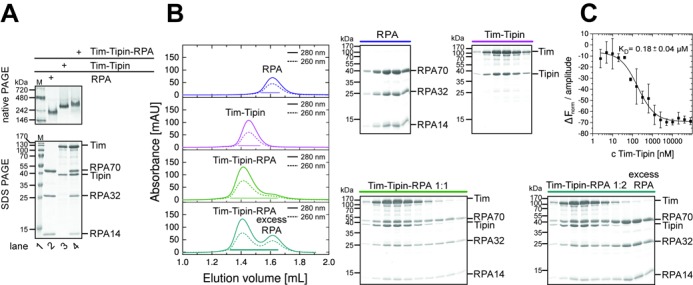
Tim-Tipin-RPA forms a 1:1:1 complex. (A) Native PAGE analysis reveals a homogeneous RPA (lane 2), Tim-Tipin (lane 3) and Tim-Tipin-RPA complex (lane 4). The assembly of the protein complexes was analyzed on Coomassie stained native gels (top) and by SDS-PAGE (bottom). M, molecular weight marker in kDa (lane 1). (B) SEC shows 1:1:1 complex formation of Tim-Tipin and RPA. Trimeric RPA and dimeric Tim-Tipin complexes ran as a single peak in SEC (blue and magenta). The 1:1 molar ratio mixture of RPA and Tim-Tipin eluted essentially as a single peak and shifted to higher molecular weight (green). Adding RPA at one molar excess to Tim-Tipin, showed an additional peak with the excess of RPA (cyan). The elution profile was visualized by UV absorbance at 280 nm (solid) and 260 nm (dashed). Peak fractions are indicated with lines and the corresponding Coomassie stained SDS-gels are shown (right). (C) MST titration of RPA with Tim-Tipin shows a *K*_D_ of 0.18 μM.

These individually purified proteins were mixed and the complex formation of Tim-Tipin-RPA was carried out. GST-pull-down assays confirmed the previously reported interaction between the Tim-Tipin complex and RPA (Supplementary Figure S1E) (3,14,37). The Tim-Tipin-RPA mixture showed a homogenic complex formation by native polyacrylamide gel electrophoresis (Figure 1A). To determine the stoichiometry of the components in the Tim-Tipin-RPA complex, analytical SEC and SLS were performed (Figure 1B, Table 1). The SEC profile of the mixture of RPA and Tim-Tipin at a 1:1 molar ratio was shifted to higher molecular weight compared to the individual components (Figure 1B, Table 1). Further SLS analysis showed the molecular weights of 273 ± 31 kDa for Tim-Tipin-RPA, 164 ± 12 kDa for Tim-Tipin and 106 ± 6 kDa for RPA, which is comparable with the theoretical molecular weights based on the amino acid sequence (Supplementary Figure S2A, Table 1). In addition, we performed sucrose-gradient centrifugation to estimate the size of Tim-Tipin. Tim-Tipin (theoretical value 162 kDa) sedimented at the same position as γ-globulin (158 kDa) (Supplementary Figure S2B), further confirming the molecular weight of the Tim-Tipin complex.

**Table 1.** Elution volumes, experimental and theoretical molecular weights of RPA, Tim-Tipin and Tim-Tipin-RPA

Protein complex	Elution volume [ml]	Experimental^SEC#^ molecular weight [kDa]	Experimental^SLS&^ molecular weight [kDa]	Experimental^SG*^ molecular weight [kDa]	Theoretical^§^ molecular weight [kDa]
RPA	1.63 ± 0.02	110	106 ± 6	N.D.	96.2
Tim-Tipin	1.46 ± 0.01	321	164 ± 12	158	162.3
Tim-Tipin-RPA	1.43 ± 0.02	388	273 ± 31	N.D.	258.5

^#^Molecular weights derived from SEC were estimated based on the comparison with molecular mass standard proteins.

^&^Molecular weights determined by SLS.

*Molecular weight determined by sucrose-gradient sedimentation assay (SG).

^§^Molecular weights calculated based on the amino acid sequence.

N.D. = not determined.

The addition of RPA to Tim-Tipin in a 2-fold excess showed an additional peak of RPA (cyan, Figure 1B), which was not incorporated into the complex. From these collective observations, we conclude that Tim-Tipin and the RPA trimer form a complex with a 1:1:1 stoichiometry. The binding affinity (*K*_D_) of Tim-Tipin to RPA was measured to be 0.18 ± 0.04 μM using MST (Figure 1C).

### Electron microscopic reconstruction of the Tim-Tipin-RPA complex.

To obtain insights into the architecture of the Tim-Tipin-RPA complex, we observed the complex by negative stain EM. We employed the GraFix method to prepare stable complexes for single-particle EM analysis. During the GraFix preparation, the complexes undergo a weak, gradual chemical fixation with glutaraldehyde while being purified by sedimentation in a sucrose gradient (42) (Supplementary Figure S3A–F). The SDS-PAGE of the cross-linked complexes (Supplementary Figure S3) indicated that fraction F10 corresponds best to the molecular weight of Tim-Tipin-RPA. For further analysis of fractions F8, F10 and F12, quantitative mass spectrometry (ESI-MS/MS and iBAQ) (46) was performed. F10 gave a 1:1 (Tim-Tipin to RPA) stoichiometry, while F8 and F12 did not show stoichiometric ratios (Supplementary Table S1). Based on these observations, fraction F10 was chosen for EM analysis. For RPA and Tim-Tipin, fractions F9 migrated close to the molecular weight of 162 kDa (Tim-Tipin) and 96 kDa (RPA) on SDS-PAGE.

**Figure 2.**
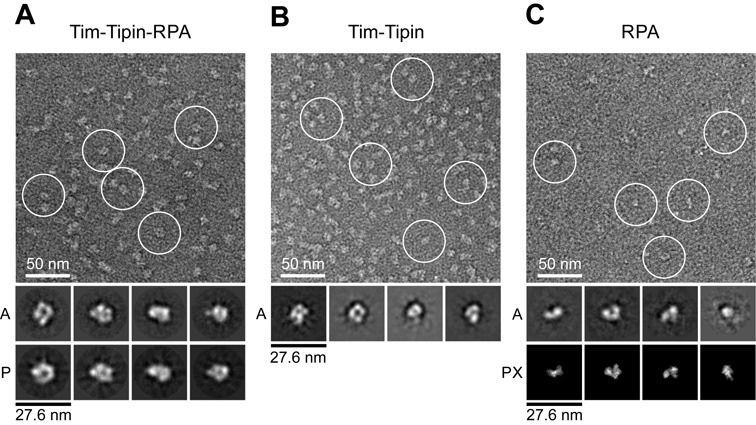
EM analysis of Tim-Tipin-RPA, Tim-Tipin and RPA. (A) Negative stain image of cross-linked Tim-Tipin-RPA (top). Examples of Tim-Tipin-RPA complex are marked with white circles. A, representative 2D class averages; P, reprojections of the 3D reconstruction. (B) Negative stain image of cross-linked Tim-Tipin. The image indicates that the main feature of the Tim-Tipin-RPA complex (Figure 2A) is given by Tim-Tipin. A, examples of 2D class averages. (C) Negative stain image of cross-linked RPA, showing a rod and horse shoe-shape structure of RPA. 2D class averages of RPA (marked with ‘A’) show comparable features to projections of the crystal structure of RPA bound to 32 nt ssDNA (PDB 4GNX) (marked with ‘PX’) (20).

The resulting specimens (Figure 2A) were homogeneous and the image contrast was increased by chemical fixation of complexes compared to untreated samples (Supplementary Figure S4A). The electron micrographs showed a stable, monodisperse complex formation of Tim-Tipin-RPA and no aggregates were found for the cross-linked sample (Figure 2A), while the untreated sample displayed slight heterogeneity and additional small densities, indicating partial dissociation of the complex (Supplementary Figure S4A). Nevertheless, 2D averages of 13 311 selected particles of the GraFix sample (Figure 2A) and 1914 selected intact particles of the untreated sample showed comparable features (Supplementary Figure S4B, top row).

The class averages of the selected particles showed well-defined features of various views with a uniform size of ∼150 Å indicating that the complex has an overall globular architecture. We recognized a core ring-like shape, a U-shape and particles displaying four different globular domains (Figure 2A).

We next performed a 3D reconstruction of the Tim-Tipin-RPA complex. To gain an initial 3D map (Supplementary Figure S4C), the RCT method (53) was used and the initial model was further refined using the untilted data set (Supplementary Figure S5A). The refined reconstruction comprises an overall globular shape with maximal dimensions of ∼150 Å × 120 Å × 120 Å at ∼23 Å resolution (according to the FSC = 0.5 criterion) (Supplementary Figure S5B).

To gain further insights into the structural compositions of the Tim-Tipin-RPA complex, we performed negative stain EM of Tim-Tipin or RPA alone. 2D class averages showed specific shapes of Tim-Tipin and RPA (Figure 2B and C). Tim-Tipin displayed a ring-like shape with ∼100 Å × 100 Å dimensions (Figure 2B). RPA alone revealed a rod-like shape (∼90 Å × 55 Å) and a smaller U-shape (∼90 Å × 80 Å) structure with 2 or 3 globular densities, which is in good agreement with the dimensions of reprojections of the crystal structure of the RPA trimer bound to 32 nt ssDNA (20) (Figure 2C (PX)). The comparison of the 2D class averages of Tim-Tipin-RPA, Tim-Tipin and RPA only showed that Tim-Tipin is the component responsible for the characteristic features of the Tim-Tipin-RPA complex.

**Figure 3.**
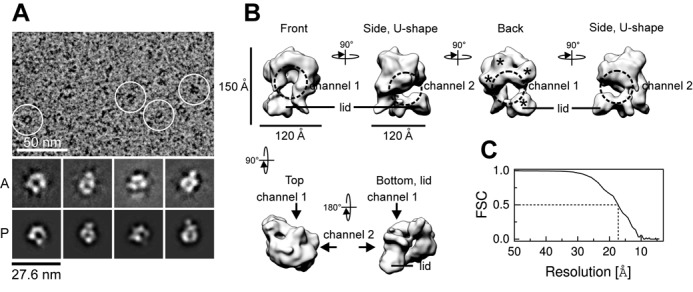
Cryo-EM analysis of the Tim-Tipin-RPA complex. (A) Representative image of vitrified Tim-Tipin-RPA complex. Examples of individual particles are circled in white. A, representative 2D class averages. P, projections of the 3D reconstruction. (B) Cryo-EM reconstruction of the Tim-Tipin-RPA complex using the negative stain 3D model (Supplementary Figure S5B) as initial reference and showing different views. The models are related by rotation around the *y*- and *x*-axis as indicated. The major density in the reconstruction displays a ring-like structure (front, dashed circle) containing a ∼30-Å-wide channel (channel 1) that is closed by a lid density at the bottom (lid). A 90° rotation around *y* unveiled a connection between the major ring-like density and the lid, forming a U-like feature with a second channel (channel 2, dashed circle). The view from the back reveals four different domains (marked by stars). (C) FSC plot for the estimation of the resolution obtained from FSC curves between two half-reconstructions. For obtaining the FSC curve, selected particles were divided into two and two resulting reconstructions were individually calculated following the method suggested by Scheres *et al.* (54).

Next, we analyzed the vitrified Tim-Tipin-RPA complex under cryo-EM (Figure 3 and Supplementary Figure S4). 3D reconstruction was performed using the 3D model of the negative-stained samples as a reference (Supplementary Figure S5B). The final map at 17 Å resolution (Figure 3C) shows a ring-like density forming a channel (channel 1) covered by a lid at the bottom (lid) (Figure 3B, front). Both domains are connected forming a U-shaped density, which is referred to as channel 2. The view at the back side of the 3D map shows four distinctive domains (back, stars). The comparison of the reprojections (P) of the 3D map and the 2D averages (A) (Figure 3A and Supplementary Figure S4B) shows good consistency.

**Figure 4.**
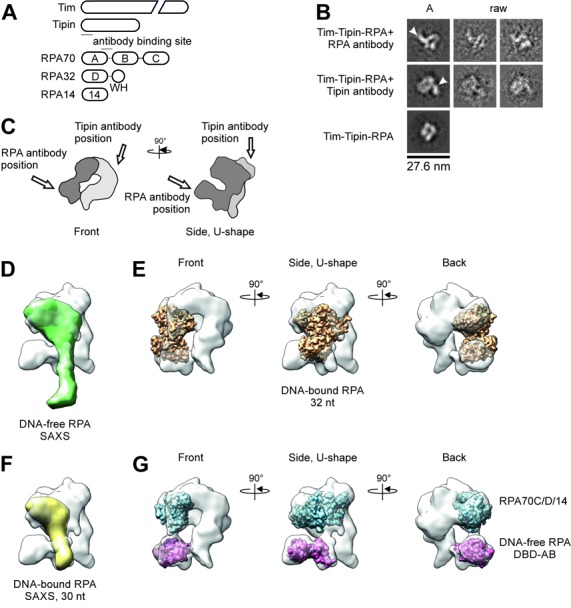
Antibody labeling of Tim-Tipin-RPA using negative stain EM and docking analysis. (A) Scheme of Tim-Tipin-RPA domain organization together with the antibody binding site within RPA70 and Tipin (solid line). (B) Different views of Tim-Tipin-RPA with an antibody against the RPA70 subunit (first row), against the N-terminus of Tipin (second row) and Tim-Tipin-RPA without antibody (third row). The RPA antibody binds sideward (marked by an arrowhead) to the ring-like density, the Tipin antibody binds on top right of the ring-like density (arrowhead). A, representative 2D class averages. raw, representative raw images. (C) Schematic representation of Tim-Tipin-RPA in two different views showing the putative localization of RPA within the model corresponding to a volume of ∼96 kDa. The putative RPA density is represented as dark gray area. The localization of the Tipin and RPA antibody is marked by an arrow. (D)–(G) Docking analysis of RPA models into the Tim-Tipin-RPA 3D reconstruction shown as transparent surface representation. RPA models: (D) SAXS model of DNA-free RPA (BioIsis ID RPADCP). (E) Crystal structure of RPA bound to 32 nt ssDNA (PDB 4GNX). (F) SAXS model of 30 nt DNA-bound RPA (BioIsis DBC30Y). (G) Crystal structure of the RPA trimerization core (blue, PDB 1L1O) and crystal structure of DNA-free RPA DBD-A and -B (magenta, PDB 1FGU chain A). In (E) and (G) three different views are shown related by 90° rotation as indicated.

To map the location of RPA and Tipin in the structure of the Tim-Tipin-RPA complex, we performed antibody labeling (Figure 4A–C). We used an available polyclonal antibody (Tipin antibody sc-160865) against the N-terminus of Tipin as well as a monoclonal antibody (RPA 70 kDa subunit antibody sc-166023) against the RPA DBD-A (Figure 4A), which does not directly interact with Tim-Tipin according to our SEC analysis (Supplementary Figure S6B). 2D class averages of the RPA-antibody labeled Tim-Tipin-RPA complex displayed an additional density at the side of the U-like shape of the 2D class average (Figure 4B, arrowhead) for RPA. The corresponding position in the 3D reconstruction appears to be at the channel 2 sitting at the left-bottom side of the ring-like feature of Tim-Tipin-RPA (Figure 4C, dark gray). The lid domain together with a part of the U-shape domain likely accommodates RPA. Antibody labeling suggests that the RPA70 DBD-A and -B correspond to the lid domain, while RPA70 DBD-C, RPA32 DBD-D and RPA14 could be located in the upper part of the opened U-shape density. Notably, the 2D class averages of the complex labeled with a Tipin antibody showed an additional density at the top right of the U-like shape density (Figure 4B, arrowhead and C, light gray). The top of the ring-like density comprises the RPA32WH-Tipin-C-Terminus interface, consistent with previous protein–protein interaction studies (3,37). Furthermore, our results suggest that the right part of the ring-like density in our reconstruction most likely harbors Tim.

### RPA employs a compact conformation within the Tim-Tipin-RPA complex

Recently, a small angle X-ray scattering (SAXS) analysis was performed on RPA in its different DNA-binding modes (19). DNA-free RPA was found to adopt an extended conformation and displays large inter-domain flexibility. Upon DNA-binding, RPA undergoes two transitions: The first transition happens when it binds to 10 nt ssDNA, compacting DBD-A and -B, and the second transition occurs upon binding to >20 nt ssDNA, further compacting the trimerization core (RPA70C/32D/14) and DBD-A and -B into a U-shape conformation (19). The crystal structure of RPA bound to 32 nt ssDNA (20) (PDB 4GNX) revealed an even more compact horse shoe-like RPA conformation. To find out which of these RPA conformations is most consistent with our EM model, we compared the RPA structure in the Tim-Tipin-RPA 3D reconstruction to the SAXS models and RPA crystal structures.

Docking the SAXS model of DNA-free RPA (19) (BioIsis ID RPADCP) into the Tim-Tipin-RPA 3D reconstruction using rigid-body fitting did not allow any reasonable placement of the SAXS model, leaving nearly half of the model outside of the envelope (Figure 4D). The crystal structure of RPA bound to 32 nt ssDNA (20) (PDB 4GNX) and the corresponding SAXS model (19) (BioIsis ID DBC30Y) (Figure 4E and F), both fit to the cryo-EM reconstruction. Further, by taking the flexibility of the RPA domains into consideration, individual docking of the crystal structure of DNA-free RPA70 DBD-AB (PDB 1FGU chainA) and the RPA trimerization core (PDB 1L1O) into the Tim-Tipin-RPA 3D reconstruction was performed and this gave the best fitting (Figure 4G).

From this docking analysis and our antibody labeling (Figure 4), we conclude that our 3D reconstruction accommodates RPA in a rather compact mode revealing a horse shoe-like conformation as reported by Fan *et al.* and Brosey *et al.*, while the extended conformation of RPA (171 Å in length) could not fit to the EM reconstruction.

It should be noted that the comparison of the 2D class averages and the 3D reconstruction of the GraFix cross-linked and SEC-reconstituted specimens showed no detectable difference on the structural arrangement of the RPA conformation within the complex (Supplementary Figures S4B and S5C), indicating that the fixation of the conformation occurs via the binding of Tim-Tipin rather than the chemical cross-linking.

### Tim-Tipin-RPA complex formation requires all three RPA subunits

Tipin has been reported to interact with the RPA32 C-terminal WH domain (3,14,37). However, in our SEC experiments we only detected a weak interaction of Tim-Tipin with the RPA32 WH domain (Supplementary Figure S6A). To further investigate which components of the trimeric RPA complex facilitate complex formation, we tested the interaction of Tim-Tipin with various RPA subcomplexes using SEC (Supplementary Figure S6).

The SEC profile of the mixture of Tim-Tipin and RPA70 DBD-A and -B (Supplementary Figure S6B) or RPA32/14 (Supplementary Figure S6C) showed no sign of interactions, as the components are eluted without influencing each other. On the other hand, the trimeric RPA70DBD-C/32-WH/14 complex (lacking RPA70 DBD-A and -B) showed a weaker interaction with Tim-Tipin (Supplementary Figure S6D) compared to the RPA70/32/14 complex containing all DBD-A, -B, -C and -D (Figure 1B). This suggests that the RPA70 DBD-A and DBD-B do not directly bind to Tim-Tipin but play an indirect role in stabilizing the Tim-Tipin-RPA complex.

Altogether, it appears that individual RPA subunits are not sufficient for Tim-Tipin-RPA complex formation. Rather, the trimeric RPA complex containing all four DBDs A–D is required, likely to support conformational arrangements of RPA. This result agrees with our cryo-EM reconstruction of the Tim-Tipin-RPA complex, which suggests that trimeric RPA in its compact U-shaped conformation provides a molecular saddle for Tim-Tipin recruitment, whose structural integrity depends on the presence of all four DBDs (A–D).

### The Tim-Tipin-RPA complex dissociates when RPA binds ssDNA in its 8 nt binding mode

While our EM structure suggests that RPA in the Tim-Tipin-RPA complex is fixed in a compact conformation, conformational changes of RPA are known to be coupled to the length of ssDNA and/or the number of RPA molecules bound to ssDNAs (19,20,28,30,33). To investigate the effect of the RPA conformation on the stability and ssDNA binding activity of the Tim-Tipin-RPA complex, we analyzed the interaction of the Tim-Tipin-RPA complex with ssDNA oligonucleotides of various lengths (60, 31 and 14 nt) at different protein:ssDNA stoichiometric ratios using SEC and EMSAs. For comparison, we also examined the binding of RPA and the Tim-Tipin complex to various ssDNA substrates under our experimental conditions.

**Figure 5.**
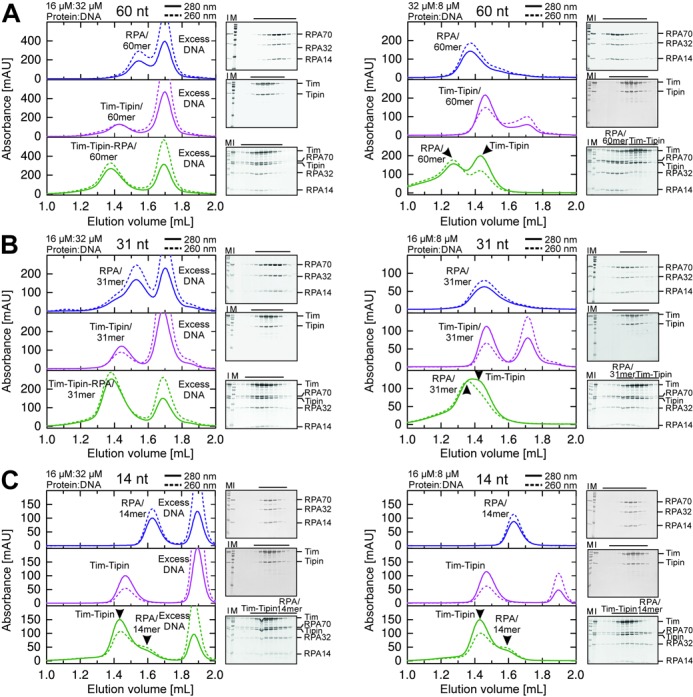
SEC analysis of RPA, Tim-Tipin and Tim-Tipin-RPA binding to ssDNA substrates. Size exclusion chromatograms of RPA (blue), Tim-Tipin (magenta) and Tim-Tipin-RPA (green) with 60 nt (A), 31 nt (B) and 14 nt (C) ssDNA and SDS-gels stained with Coomassie blue displaying the SEC protein peak fractions (black line). M, protein marker. I, protein mixture injected on SEC. Solid line: UV absorbance at 280 nm. Dashed line: UV absorbance at 260 nm. For 60 nt (A, left) and 31 nt (B, left) ssDNA, an excess of DNA resulted in an association of RPA, Tim-Tipin and Tim-Tipin-RPA with ssDNA. Accumulation of RPA on 60 nt (A, right) and 31 nt (B, right) ssDNA resulted in a breaking of the complex into DNA-free Tim-Tipin and DNA-bound RPA. Note that 14 nt ssDNA disassembles the Tim-Tipin-RPA complex and does not bind to Tim-Tipin regardless of experimental conditions (C).

**Figure 6.**
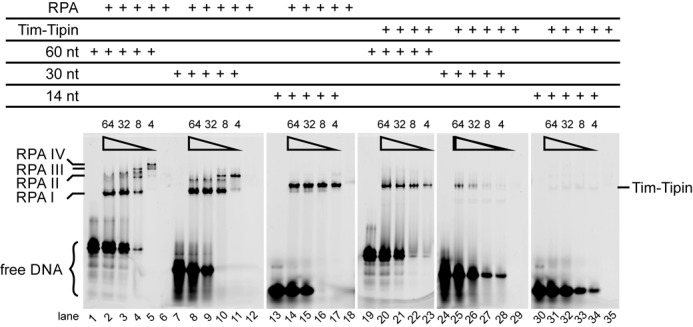
Binding of RPA and Tim-Tipin to ssDNA substrates. EMSA using a 60, 30 and 14 nt 5′-FAM labeled ssDNA substrate. 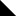 indicates decreasing amounts of the ssDNA substrate added (total concentration of 64, 32, 8 and 4 μM). The protein concentration was kept constant at 16 μM. Up to 4 RPAs bind to 60 nt ssDNA (RPA I-IV, lanes 1–6), 2 RPA to 30 nt (RPA I-II, lanes 7–12) and 1 RPA binds to 14 nt ssDNA (RPA I, lanes 13–18). Tim-Tipin binds as a monomer to the ssDNA substrates and shows the highest preference for 60 nt ssDNA (Tim-Tipin, lanes 19–23) followed by 30 nt ssDNA (lanes 24–29). No binding of Tim-Tipin to 14 nt ssDNA is observed (lanes 30–35). Unbound DNA is marked as free DNA.

When RPA was pre-incubated with excess 60 or 31 nt ssDNA, respectively, the SEC peak was shifted to higher molecular weight (Table 2 and Figure 5A and B, left) compared to DNA-free RPA (Figure 1B) and showed an increased 260/280 ratio of 1.57 ± 0.05 and 1.46 ± 0.01 for 60 and 31 nt ssDNA, respectively, indicating that DNA is bound to RPA. In EMSA analyses these complexes correspond to one molecule of RPA (110 kDa) bound to one molecule of 60 nt (18.4 kDa) or 31 nt ssDNA (9.5 kDa) (Figure 6, lanes 3 and 9).

**Table 2.** Elution volumes, 260/280 ratio, experimental and theoretical molecular weights of RPA, Tim-Tipin (TTP) and Tim-Tipin-RPA in presence of ssDNA substrates

DNA substrate/ Protein:ssDNA [μM]	Protein mixture/ DNA substrate	Elution volume* [ml]	260/280*	Experimental^SEC#^ molecular weight [kDa]	Theoretical^§^ molecular weight [kDa]
	RPA	1.63 ± 0.02	0.63 ± 0.02	110	96.2
	Tim-Tipin	1.46 ± 0.01	0.55 ± 0.01	321	162.3
	Tim-Tipin-RPA	1.43 ± 0.02	0.56 ± 0.01	388	258.5
					
60 nt	RPA	1.54 ± 0.00	1.57 ± 0.05	194	114.6
16:32	Tim-Tipin	1.44 ± 0.01	0.98 ± 0.14	364	180.7
	Tim-Tipin-RPA	1.39 ± 0.01	1.20 ± 0.04	498	276.9
60 nt	RPA	1.38 ± 0.01	1.32 ± 0.03	531	403.2
32:8	Tim-Tipin	1.47 ± 0.01	0.74 ± 0.06	301	180.7
	Tim-Tipin-RPA	1.44 ± 0.01 (TTP)	0.66 ± 0.05	364	
		1.33 ± 0.05 (RPA)	1.13 ± 0.04	726	
31 nt	RPA	1.55 ± 0.03	1.46 ± 0.01	182	105.7
16:32	Tim-Tipin	1.46 ± 0.02	0.74 ± 0.01	321	171.8
	Tim-Tipin-RPA	1.40 ± 0.03	1.11 ± 0.04	468	268.0
31 nt	RPA	1.48 ± 0.02	1.28 ± 0.05	283	201.9
16:8	Tim-Tipin	1.47 ± 0.01	0.57 ± 0.02	301	171.8
	Tim-Tipin-RPA	1.41 ± 0.03 (TTP)	0.87 ± 0.0	439	
		1.38 ± 0.03 (RPA)	0.98 ± 0.01	531	
14 nt	RPA	1.63 ± 0.0	1.47 ± 0.24	110	100.5
16:32	Tim-Tipin	1.48 ± 0.01	0.54 ± 0.02	283	166.6
	Tim-Tipin-RPA	1.45 ± 0.02 (TTP)	0.66 ± 0.08	342	
		1.62 ± 0.01 (RPA)	1.27 ± 0.05	117	
14 nt	RPA	1.63 ± 0.0	1.27 ± 0.06	110	100.5
16:8	Tim-Tipin	1.48 ± 0.01	0.54 ± 0.0	283	166.6
	Tim-Tipin-RPA	1.45 ± 0.02 (TTP)	0.64 ± 0.05	342	
		1.62 ± 0.01 (RPA)	1.22 ± 0.04	117	
ssDNA only	60 nt	1.70	1.82	64	18.4
0:16	31 nt	1.70	1.76	64	9.5
	14 nt	1.88	2.34	21	4.3
	Thyroglobulin	1.32 ± 0.02*			670
	γ-globulin	1.63 ± 0.03*			158
	Ovalbumin	1.76 ± 0.01*			44
	Myoglobin	1.91 ± 0.02*			17
	Vitamin B_12_	2.15 ± 0.02*			1.35

^#^Molecular weights estimated based on the comparison with molecular mass standard proteins.

^§^Molecular weights calculated based on the amino acid sequence.

* Average numbers and standard deviations of at least two independent measurements are shown.

As the ssDNA concentration was reduced to substoichiometric amounts (protein:DNA, 32 μM:8 μM or 16 μM:8 μM), the SEC peak of RPA further shifted to higher molecular weight (Table 2 and Figure 5A and B, right). The peak fractions still contain ssDNA (260/280 = 1.32 ± 0.03, 1.28 ± 0.05), showing the accumulation of the excess RPAs on ssDNA (Figure 5A and B, right). Corresponding EMSA analysis (Figure 6) showed a stepwise accumulation of excess RPA on ssDNA, where the degree of accumulation depends on the length of ssDNA. We determined the stoichiometry of RPA to ssDNAs at the saturation level to be two (RPA II) for 30 nt (Figure 6, lane 11) and four (RPA IV) for 60 nt (Figure 6, lane 5). In contrast, we detected one RPA molecule (RPA I) for the 14 nt ssDNA substrate (Figure 6, lanes 13–18). Note that under the conditions where either the ssDNA is short (here 14 nt), or RPAs accumulate on ssDNA (here substoichiometric DNA amounts), RPA is reported to employ the 8 nt binding mode (17,30,33,55,56). We also observed faint densities smeared below the main bands (Figure 6, lanes 14–16, Supplementary Figure S7A, lanes 7–9, marked with star). Direct comparisons of the EMSA profiles of RPA with 8, 14, 30 or 60 nt ssDNA in various concentrations showed that these faint bands migrated at a comparable position as RPA in the 30 nt mode (Supplementary Figure S7A, lane 5), indicating that in the presence of 14 nt ssDNA, a small population of RPA adopts the 30 nt binding mode.

Note that this faint densities below the main band were not observed with 8 nt (Supplementary Figure S7A, lanes 11–13). In the presence of 8 nt ssDNA, RPA migrated less than 14 nt ssDNA, possibly due to different charges of the ssDNA oligonucleotides.

In addition, we occasionally observed an upper faint band (Figure 6, lane 17, Supplementary Figure S7A, lane 11) representing likely a stochastically happening artifact.

Next, we examined the ssDNA binding characteristics of Tim-Tipin. The SEC peak of Tim-Tipin with an excess of 60 and 31 nt ssDNA showed only a small shift to higher molecular weight (Table 2 and Figure 5A and B, left) compared to DNA-free Tim-Tipin (Figure 1B). These peak positions were virtually indistinguishable, presumably due to the detection limit of the experimental system. However, increased 260/280 ratio was detected for 60 nt ssDNA (0.98 ± 0.14) and for 31 nt ssDNA (0.74 ± 0.01) (Figure 5A and B, left) compared to DNA-free Tim-Tipin (260/280 = 0.55 ± 0.01, Figure 1B), indicating that ssDNA was partially bound to Tim-Tipin. Consistent with our SEC results, we observed a band shift of the 30 and 60 nt ssDNAs to the position of Tim-Tipin-DNA complex in EMSA (Figure 6, lanes 19–23 for 60 nt; lanes 24–29 for 30 nt). This effect was even more pronounced with 60 nt ssDNA than with 30 nt ssDNA. From the EMSA profile, the stoichiometry of Tim-Tipin to ssDNAs was determined to be 1:1. Notably, we did not detect any binding of Tim-Tipin to 14 nt ssDNA neither in EMSA (Figure 6, lanes 30–35) nor in SEC analyses (Figure 5C, 260/280 = 0.54 ± 0.02 (left) 0.54 ± 0.0 (right)). Additionally, binding isotherms of Tim-Tipin to 60 and 30 nt ssDNA obtained from FA (Supplementary Figure S7B) quantified that Tim-Tipin binds to 30 nt ssDNA with a *K*_D_ of 1.7 ± 0.2 μM and to 60 nt ssDNA with a *K*_D_ of 0.29 ± 0.01 μM. The weak affinity is in line with the partial binding of ssDNA in SEC.

**Figure 7.**
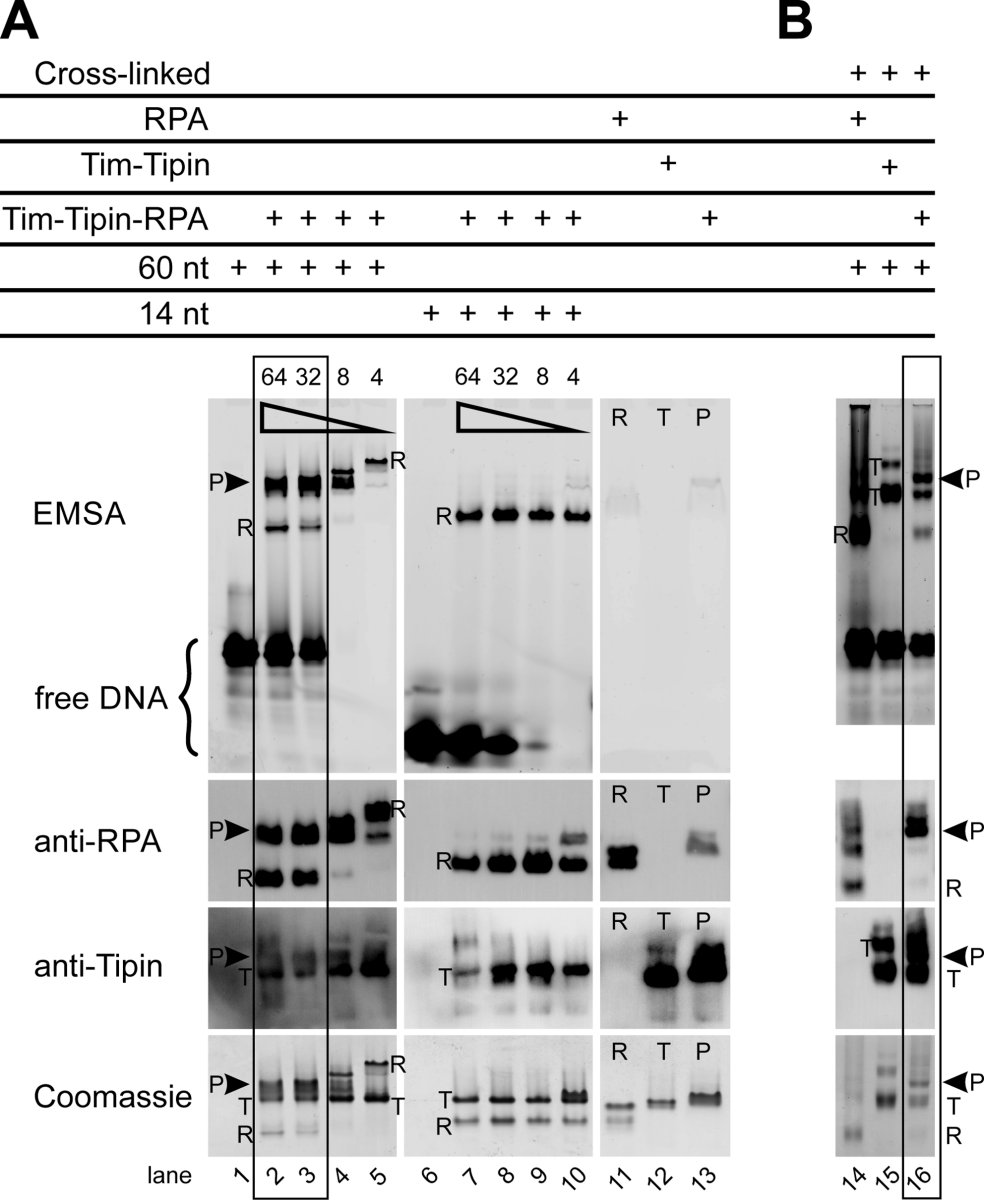
Binding of Tim-Tipin-RPA to ssDNA substrates. (A) EMSA using 60 and 14 nt 5′-FAM labeled ssDNA substrate. The protein-ssDNA complexes were visualized by fluorescence imaging (EMSA), immunoblot analysis using an anti-RPA70 and anti-Tipin primary antibody and Coomassie stain. In the presence of long excess ssDNA significant amounts of intact Tim-Tipin-RPA-ssDNA complex were detected as indicated by the left square. The presence of substoichiometric amounts of long ssDNA (lanes 4 and 5) and short ssDNA (lanes 6–10) lead to dissociation of the Tim-Tipin-RPA-ssDNA complex into DNA-free Tim-Tipin and DNA-bound RPA. The total ssDNA concentration was varied from 64, 32, 8 to 4 μM as indicated by (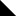). The Tim-Tipin-RPA concentration was kept constant at 16 μM. R, RPA; T, Tim-Tipin; P, pentameric Tim-Tipin-RPA. Lanes 11–13 show the DNA-free proteins. (B) EMSA after cross-linking the complex. Double excess amounts of 60 nt 5′-FAM labeled ssDNA and RPA (3.12 μM:1.56 μM RPA), Tim-Tipin (1.84 μM:0.92 μM Tim-Tipin) and Tim-Tipin-RPA (1.2 μM:0.6 μM Tim-Tipin-RPA) are used. After incubation with the DNA substrate, the protein complexes were cross-linked with glutaraldehyde, analyzed by EMSA and visualized as in (A).

Finally, we characterized the binding of the Tim-Tipin-RPA complex to various lengths and concentrations of ssDNAs using SEC. Tim-Tipin-RPA mixed with excess 60 and 31 nt ssDNA eluted at higher molecular weight (Figure 5A and B, left) than Tim-Tipin-RPA without DNA (Figure 1B) and displayed an increased 260/280 ratio of 1.20 ± 0.04 (60 nt) and 1.11 ± 0.04 (31 nt) (Table 2), implying ssDNA binding of the intact Tim-Tipin-RPA complex with a RPA conformation likely resembling its compact 30 nt binding mode. Corresponding EMSA analysis (Figure 7) showed the binding of ssDNA to the complex (Figure 7A, lanes 2 and 3, arrowhead marked as P), although a partial dissociation of the Tim-Tipin-RPA-ssDNA complex was also observed.

Interestingly, in the presence of substoichiometric amounts of 30 and 60 nt ssDNA (Tim-Tipin-RPA complex in excess), we observed an entire dissociation of the Tim-Tipin-RPA-ssDNA complex to DNA-free Tim-Tipin and ssDNA-associated RPAs (Figure 5A and B, right, Figure 7A, lanes 4 and 5). Concomitantly, the EMSA showed RPA accumulation on ssDNA with up to 4 RPAs for 60 nt (Figure 7A, lane 5), which is in line with the EMSA of RPA-ssDNA alone (Figure 6, lane 5). Upon disassembly, freed RPA accumulated on ssDNA as it binds tighter than Tim-Tipin.

Further, to stabilize the complex-ssDNA formation, glutaraldehyde-cross-linking was performed with the protein-ssDNA mixture (0.6 μM Tim-Tipin-RPA, 1.2 μM ssDNA), and analyzed by EMSA (Figure 7B). Although partial dissociation was detectable, we observed significant binding of the cross-linked Tim-Tipin-RPA to ssDNA (Figure 7B, lane 16) as a supershifted band compared to RPA and Tim-Tipin alone (Figure 7B, lanes 14 and 15).

Furthermore, for the 14 nt ssDNA the Tim-Tipin-RPA complex displayed dissociation into DNA-free Tim-Tipin (260/280 = 0.66 ± 0.08 and 0.64 ± 0.05) and DNA-bound RPA employing the 8 nt mode (260/280 = 1.27 ± 0.05 and 1.22 ± 0.04) in all tested SEC conditions (Figure 5C, left, right; Table 2) as well as in EMSA (Figure 7A, lanes 7–10).

Altogether, our experiments indicate that the compact conformation of RPA stabilizes the Tim-Tipin-RPA complex formation and the change of RPA to the 8 nt mode coincides with the dissociation of the Tim-Tipin-RPA complex into DNA-bound RPA and DNA-free Tim-Tipin.

## DISCUSSION

**Figure 8.**
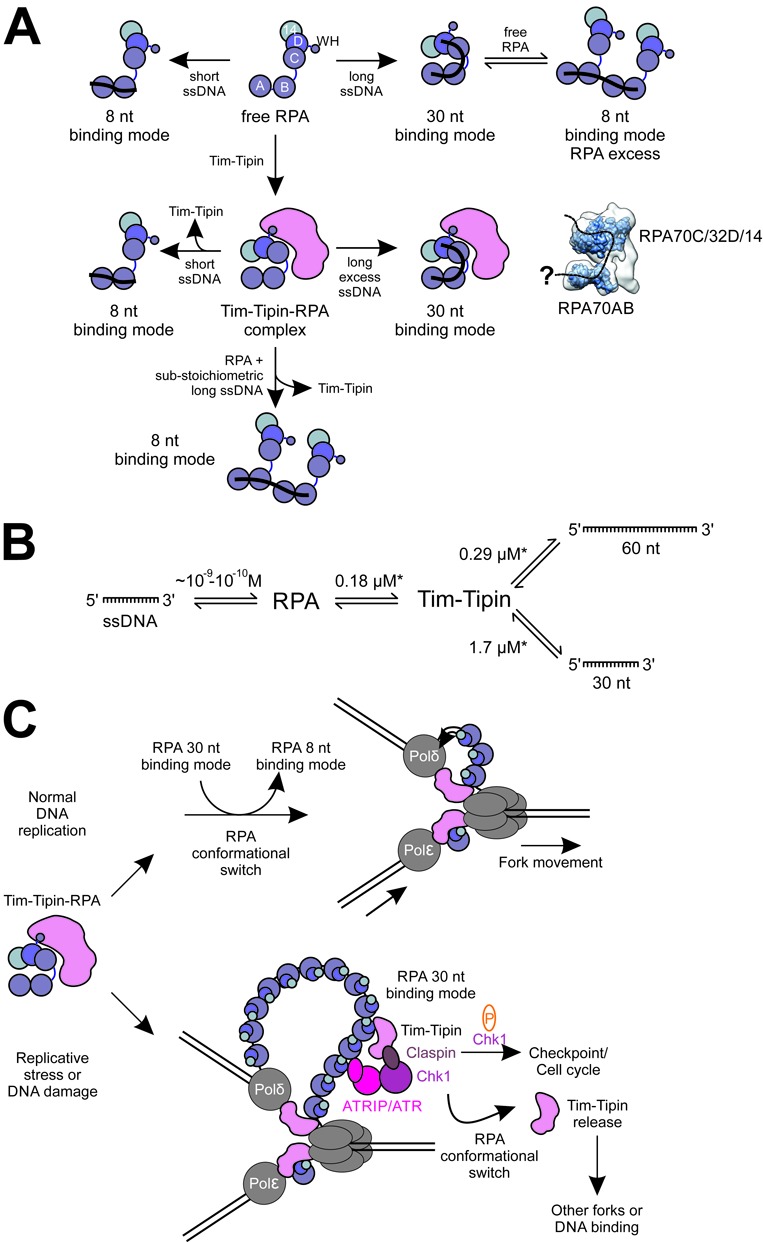
Tim-Tipin-RPA complex formation relies on ssDNA length-dependent RPA conformations. (A) Graphical scheme of the dynamic formation and disruption of the Tim-Tipin-RPA-ssDNA complex in connection with the ssDNA-length dependent conformational change of RPA. RPA shows two binding modes (8 and 30 nt binding mode), which can coexist in a dynamic equilibrium in solution (top). RPA and Tim-Tipin form a complex without and in the presence of long excess ssDNA. In the DNA-free state, the RPA conformation is fixed by Tim-Tipin into a compact mode resembling RPA's conformation in the presence of long ssDNA. The Tim-Tipin-RPA-ssDNA complex dissociates in the presence of short or substoichiometric amounts of long ssDNA, thus upon the conformational change of RPA to the 8 nt binding mode. A possible ssDNA path within our Tim-Tipin-RPA EM reconstruction is indicated as dashed line. (B) Interaction scheme of RPA and ssDNA, RPA and Tim-Tipin and Tim-Tipin and ssDNA showing the *K*_D_ determined in this study (marked by star). RPA shows a very high (nano- to subnanomolar) affinity to ssDNA in comparison to Tim-Tipin (micromolar) suggesting that Tim-Tipin binding to ssDNA might play only a minor role in the Tim-Tipin-RPA complex. (C) Hypothetical role of Tim-Tipin-RPA at the replication fork. During normal DNA replication Tim-Tipin could be bound to RPA adopting its 30 nt binding mode. After recruitment to the replication sites Tim-Tipin could get loaded onto DNA replication forks by a hand-off mechanism. During this process, RPA could perform a conformational switch adopting the 8 nt binding mode and thus releasing Tim-Tipin, which in turn is placed between the helicase and polymerase. Once placed at the fork, Tim-Tipin could then couple helicase-polymerase functions. In response to replicative stress or DNA damage accumulation of ssDNA occurs, which is covered by RPA. RPA could recruit Tim-Tipin to the accumulated ssDNA, which then becomes a part of the intra-S phase checkpoint protein complex and mediates efficient phosphorylation of Chk1 by ATR (6). Conformational change of RPA through phosphorylation (57) or interaction with other proteins could lead to Tim-Tipin release, which in turn resumes RPA-independent functions like DNA binding and helicase-polymerase coupling or associates with other forks.

Our EM and biochemical analyses of the reconstituted Tim-Tipin-RPA complex provide structural and mechanistic insights into RPA-controlled Tim-Tipin recruitment to ssDNA. RPA in the Tim-Tipin-RPA complex employs a compact conformation similar to the previously characterized complex of RPA bound to ssDNA (19,20). Our study shows that Tim-Tipin locks RPA in a conformation resembling to its 30 nt binding mode under DNA-free conditions. In the presence of ssDNA, the formation of the Tim-Tipin-RPA-ssDNA complex depends on the mode/conformation of RPA. When the length of ssDNA binding to RPA is <∼15 nt, or when more than one RPA binds to ssDNA (at high protein to DNA ratios), RPA is thought to bind to ssDNA in its less compact 8 nt mode, in which only RPA70 DBD-A and -B are involved in DNA contacts. In such conditions, we observed the disassembly of Tim-Tipin from ssDNA-bound RPA (Figures 5, 7 and 8). In contrast, in excess of >30 nt ssDNA fragments, where the most compact conformation of RPA is achieved, the Tim-Tipin complex remains associated (Figures 5 and 7, summarized in Figure 8A).

Complex formation between Tim-Tipin and RPA was previously reported to be mediated by direct interactions between Tipin and the C-terminal region of the RPA32 subunit (WH domain) (3,14,37). In our study, we observed only a weak Tim-Tipin binding to the RPA32 WH domain and no detectable interaction between Tim-Tipin and the RPA32/14 complex (Supplementary Figure S6). Since Tim-Tipin makes a complex with RPA only in the presence of all ssDNA binding RPA domains (RPA70 DBD-A, -B, -C, RPA32 DBD-D), it is conceivable that the overall horse shoe-like architecture of RPA is necessary for the relevant interaction.

In our study, we used truncated protein fragments minimally required for the reconstitution of the Tim-Tipin-RPA complex. The excluded segments are N-terminal 1–189 and 1–42 residues of RPA70 and RPA32 (RPA70N/RPA32N) and the C-terminal 1135–1197 residues of Tim. RPA70N and RPA32N are not included in the structural core of the complex (26,27). Nevertheless, we confirmed that the reconstitution of the Tim-Tipin-RPA complex including full-length RPA proteins could be successfully carried out and this Tim-Tipin-RPA(FL) complex showed a similar behavior in terms of the dependence on the stability of the complex, on ssDNA length and protein:ssDNA stoichiometry, as the truncated Tim-Tipin-RPA complex without RPA70N/RPA32N based on our SEC analysis (Supplementary Figure S8). Further, we designed a truncated Tim construct lacking the C-terminus because of a severe degradation behavior of the corresponding residues. The truncated Tim-Tipin complex is fully binding-competent to RPA, highlighting the dispensability of the C-terminal Tim fragment for complex formation.

Although the missing fragments do not affect complex formation and the investigated biochemical functions of the Tim-Tipin-RPA complex, these missing fragments may still provide further functional relevances. In the cellular environment, RPA32N is phosphorylated in a cell-cycle-dependent manner and in response to DNA damage at the first 33 residues (34,35). The introduction of negative charge to the RPA32 N-terminus leads to an intra-molecular interaction with the basic cleft of RPA70N (57). The resulting conformational changes in the RPA molecule may modulate RPA's DNA binding activity and interaction with other proteins. RPA70N and RPA32N may play a regulatory role in this sense *in vivo*. It will be insightful to know the role of RPA70N and RPA32N in the recruitment and release of Tim-Tipin in the future.

Collective observations suggest that the Tim-Tipin complex is located within the replisome between the helicase and the polymerases and plays a role in coupling DNA-unwinding and DNA-synthesis by directly affecting the catalytic activities of these enzymes (3,8–10,58).

While it is unlikely that the weak affinity of Tim-Tipin to ssDNA plays a crucial role within the Tim-Tipin-RPA complex as RPA binds to ssDNA with much higher affinity (Figure 8B), the affinity of Tim-Tipin to ssDNA may be advantageous to keep Tim-Tipin proximal to the replication machinery (Figure 8C). RPA likely mediates the recruitment of Tim-Tipin to replication forks and we could surmise Tim-Tipin may be loaded on the forks by a ‘hand-off’ mechanism.

RPA could switch the conformation from 30 to 8 nt binding mode thus releasing Tim-Tipin, which is then placed between the helicase and polymerase to couple the functions of these two machineries (Figure 8C). In this scenario, the observation that Tim-Tipin does not bind to RPA in the 8 nt mode is sensible, as the optimal location of Tim-Tipin should be at the replication fork, rather than at opened ssDNA wrapped by RPA in its 8 nt mode (Figure 8C, top).

The RPA-mediated Tim-Tipin recruitment to ssDNA may also implicate a role of the Tim-Tipin-RPA complex in the S-phase checkpoint signaling (Figure 8C, bottom). Generation of ssDNA occurs when the DNA polymerase stalls due to DNA lesions and its function is uncoupled to helicase unwinding (59,60). Through the RPA interaction Tim-Tipin may become a part of the S phase checkpoint protein complex and mediate efficient phosphorylation of Chk1 by ATR (6,13–15). The structural conformation of RPA might also impact on the Tim-Tipin recruitment/release at these sites. On the other hand, the weak binding of Tim-Tipin to ssDNA may also hint at the reported RPA-independent roles of Tim-Tipin in recognition of DNA damage (61). Further investigations are required to understand the precise role of Tim-Tipin in the response to DNA damage.

Interestingly, several studies suggested that the association of RPA-binding proteins to RPA leads to a shift of the 30 nt to the 8 nt binding mode accompanied by the dissociation of the RPA trimerization core from the ssDNA (*K*_D_ 2–10 μM) (62). These proteins include XPA, UNG2, RAD52 (25) and the SV40 Tag helicase (31,34). The interactions engaging the C-terminal region of RPA32 (WH domain), RPA70AB and RPA70N (25,34,63) have been speculated to cause a sterical hindrance, dissociating the trimerization core (RPA70C/RPA32D/RPA14) from ssDNA and furthermore may lead to the displacement of RPA from DNA. Although Tipin shares sequence similarity with XPA, UNG2 and Rad52, the Tim-Tipin-RPA complex formation on ssDNA is achieved by the 30 nt binding mode of the RPA. We speculate that, while the WH domain acts as a general sensor for the RPA-accessory components, the conformation of the RPA trimerization core also plays a certain role in the recruitment of accessory components. Regardless of the RPA binding mode, binding of these proteins to RPA and further to ssDNA occurs in a sensitive equilibrium and this may hint at the elaborated organization of the protein complexes at the DNA replication machinery. These dynamic interactions might facilitate a high turnover of the involved proteins allowing efficient and fast adaptation to incidents during DNA replication.

In summary, our observation that the formation and disruption of the Tim-Tipin-RPA-ssDNA complex is highly dynamic and modulated by the mode of RPA binding, might have implications for its role in the effective organization of the DNA replication fork. We surmise that Tim-Tipin is recruited to ssDNA by RPA to further orchestrate the arrangement of proteins involved in the replication fork and DNA repair pathway. Further investigations of Tim-Tipin in the fully assembled DNA replisome are necessary to conclusively describe the role of Tim-Tipin and the Tim-Tipin-RPA complex in DNA replication and repair.

## ACCESSION NUMBERS

The EM density map for the Tim-Tipin-RPA complex can be found at the Electron Microscopy Data Bank under accession number EMD-2789.

## SUPPLEMENTARY DATA

Supplementary Data are available at NAR Online.

SUPPLEMENTARY DATA
