# Supplementary material for: Architecture and ssDNA interaction of the Timeless-Tipin-RPA complex
Source: Nucleic Acids Res. 2014 Oct 27;42(20):12912–27. doi: 10.1093/nar/gku960 (PMC4227788; doi:10.1093/nar/gku960)
Supplement: SUPPLEMENTARY DATA [file supp_42_20_12912__index.html]

Architecture and ssDNA interaction of the Timeless-Tipin-RPA complex — Architecture and ssDNA interaction of the Timeless-Tipin-RPA complex — SUPPLEMENTARY DATA 

# Architecture and ssDNA interaction of the Timeless-Tipin-RPA complex

## SUPPLEMENTARY DATA

**Files in this Data Supplement:**

- SUPPLEMENTARY DATA
