# Supplementary material for: Architecture and ssDNA interaction of the Timeless-Tipin-RPA complex
Source: Nucleic Acids Res. 2014 Oct 27;42(20):12912–27. doi: 10.1093/nar/gku960 (PMC4227788; doi:10.1093/nar/gku960)
Supplement: SUPPLEMENTARY DATA [file supp_gku960_nar-01719-z-2014-File010.pdf]

Supplementary Information

Architecture and ssDNA interaction of the Timeless-Tipin-RPA Complex

Witosch, Justine[1], Wolf, Eva[1,2,3,*] and Mizuno, Naoko[1,*]

[1] Department of Structural Cell Biology, Max Planck Institute of Biochemistry, Am Klopferspitz 18, 82152 Martinsried, Germany

[2] Department of Physiological Chemistry and Centre for Integrated Protein Science Munich (CIPSM), Butenandt Institute, Ludwig Maximilians University of Munich, Butenandtstrasse 5, 81377 Munich, Germany

[3] Present address: Institut für allgemeine Botanik, Johannes Gutenberg-University, Johannes-von-Müller-Weg 6, 55128 Mainz, Germany and Institute of Molecular Biology (IMB) Mainz, Germany

Supplementary Figure 1

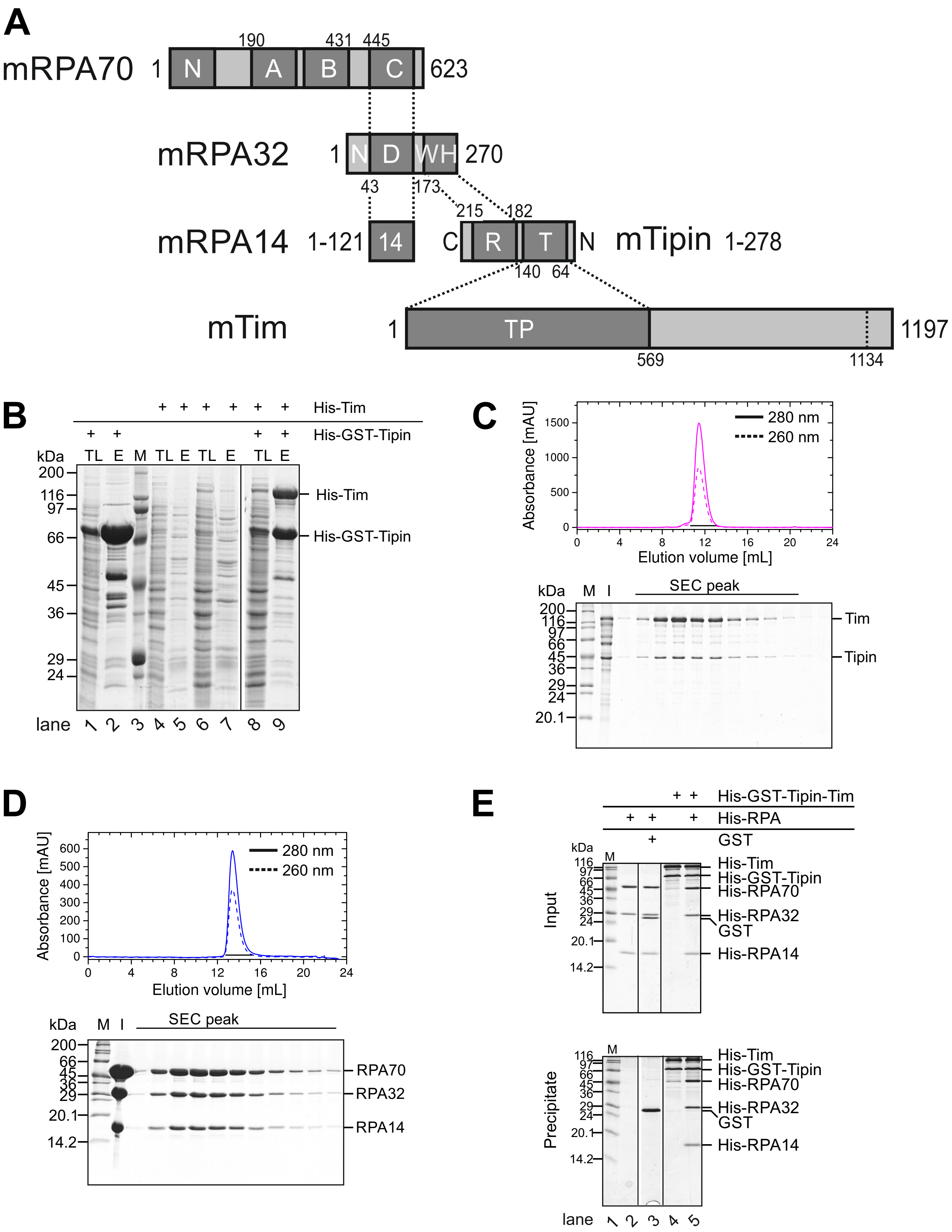

Supplementary Figure 1: Complex formation and schematic representation of mouse Timeless (Tim), Tipin and RPA

(A) Schematic representation of the domain architecture and mapped interaction sites assigned to the mouse (m) homologues Timeless (mTim), mTipin and mRPA. White N = N-terminal domain of mRPA70. A, B, C, D = DNA-binding domains (DBDs) of mRPA70 and 32. 190 = Start of DBD-A of mRPA70. 431 = End of DBD-B of mRPA70. 445 = Start of DBD-C of mRPA70. White N = N-terminal domain of mRPA32, phosphoamino acid cluster. 43 = Start of DBD-D of mRPA32. WH = RPA32 winged helix (aa 173-270). 14 = RPA14. Dashed lines mark the interaction sites between the proteins. R = Interaction site of Tipin (aa 182-215) with RPA32. T = Interaction site of Tipin (aa 64-140) with Tim. TP = interaction site of Tim (1-569) with Tipin. 1134 = End of the Tim construct used in this study. Black N = N-terminus. Black C = C-terminus.

(B) Timeless-Tipin was co-expressed as soluble complex. Recombinant expression in *E. coli* (TL, total lysate) and *in vitro* pull-down assays (E, Eluate) of His-Tim and His-GST-Tipin are visualized on Coomassie stained SDS-gels. Equal amounts of bacterial cells were lysed (TL) and soluble proteins were precipitated via their His- or GST-affinity tags using Ni-NTA (lane 4-7) or GSH sepharose (lane 1, 2, 8, 9) beads (E). His-GST-Tipin was highly expressed and precipitated via GSH beads (lane 1, 2). His-Tim was neither expressed using standard (lane 4, 5) nor codon optimized bacterial cells (lane 6, 7). Co-expression of His-Tim with His-GST-Tipin solubilized Tim forming a stable complex with Tipin on GSH beads (lane 8, 9). The cross above each lane indicates the protein component present in the expression and pull-down assay. (M) Protein marker (lane 3) with corresponding molecular weight indicated in kDa on the left.

(C) Tim and Tipin were purified together as a 1:1 complex. Size exclusion chromatogram (top) of co-purified Tim-Tipin showing a single peak elution profile visualized by UV absorbance at 280 nm and 260 nm. SDS-PAGE (bottom) shows the peak fractions marked by a line. (M) Protein marker in kDa. (I) Protein mixture injected on gel-filtration column.

(D) RPA was purified as a 1:1:1 complex containing RPA70 (DBD-A, -B, -C), RPA32 (DBD-D, WH domain) and RPA14. SEC and SDS-PAGE analysis as in (C).

(E) Tim-Tipin-RPA forms a complex. *In vitro* GST-pull-down assays incubating purified His-GST-Tipin-His-Tim complexes with His-RPA. His-RPA alone does not interact with Glutathione sepharose and free GST (lane 2 and 3). The His-GST-Tipin-His-Tim complex is precipitated on GSH-beads (lane 4) and pulls down His-RPA efficiently (lane 5). The input (top) and precipitate (bottom) were analysed on Coomassie stained SDS-gels. The cross above each lane indicates the proteins present in the input. (M) Molecular weight marker in kDa

Supplementary Figure 2

A

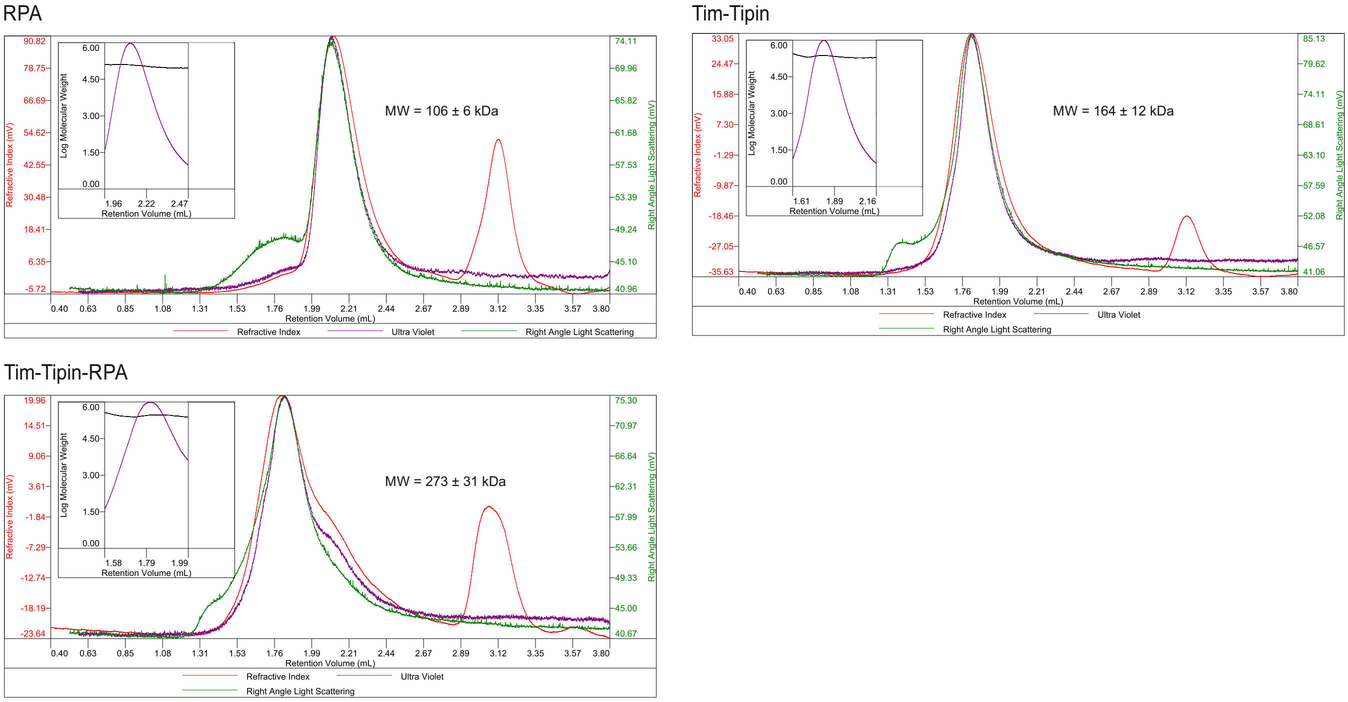

B

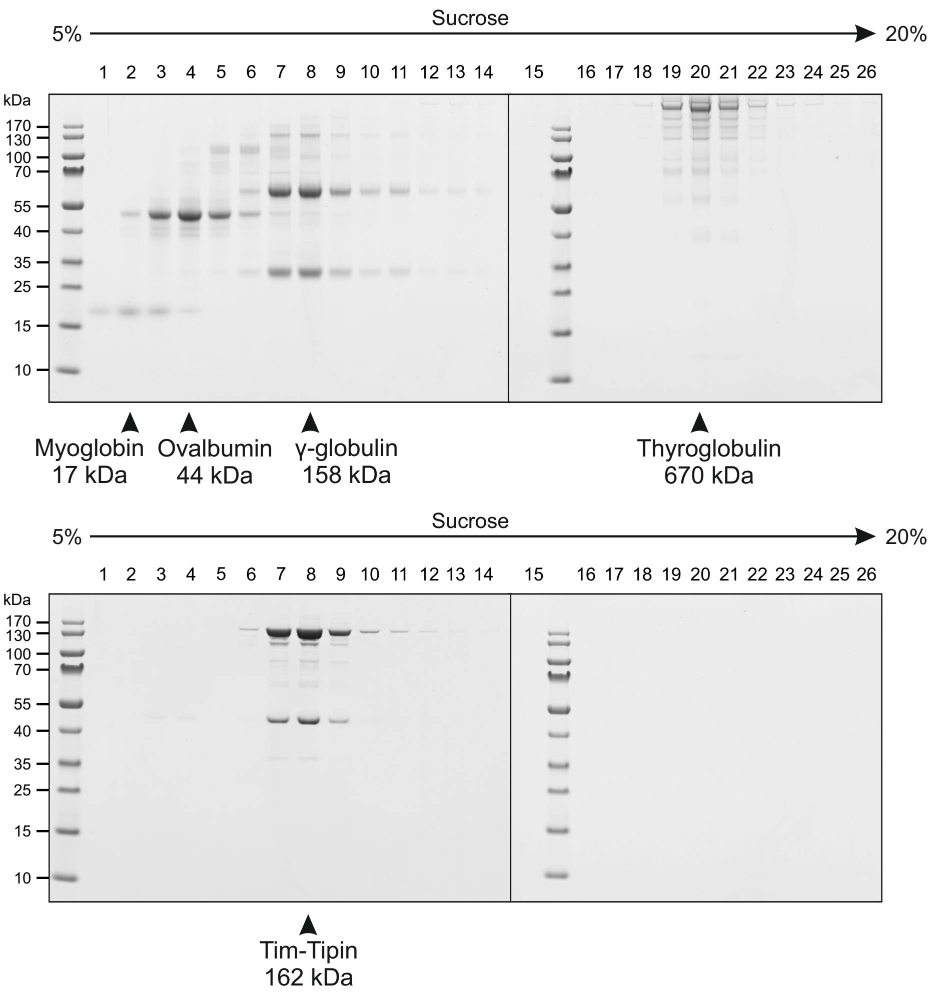

Supplementary Figure 2: Molecular weight determination of Tim-Tipin, RPA and Tim-Tipin-RPA by Static Light Scattering and gradient sedimentation.

(A) Representative static light scattering (SLS) profile of RPA, Tim-Tipin und Tim-Tipin-RPA showing UV absorbance (violet), light scattering (green) and refractive index (red) for the respective protein complex solution. The top left panels show the average molecular weight values (logarithmic scale) in the population of the peaks. It should be noted, that Tim-Tipin-RPA displays faint dissociation as indicated in the SLS analysis.

(B) Analysis of Tim-Tipin by sucrose-gradient sedimentation. The purified Tim-Tipin (162 kDa) complex was sedimented through a linear 5 to 20% (w/v) sucrose gradient and its sedimentation behavior was compared to standard proteins. Tim-Tipin showed a similar sedimentation behavior as γ-globulin. The Coomassie stained SDS-PAGE analysis of each fraction containing approximately 500 µL is shown. The used standard proteins were Myoglobin (17 kDa), Ovalbumin (44 kDa), γ-globulin (158 kDa for entire molecule of two light and two heavy chains) and Thyroglobulin (670 kDa for entire homo-dimeric molecule).

Supplementary Figure 3

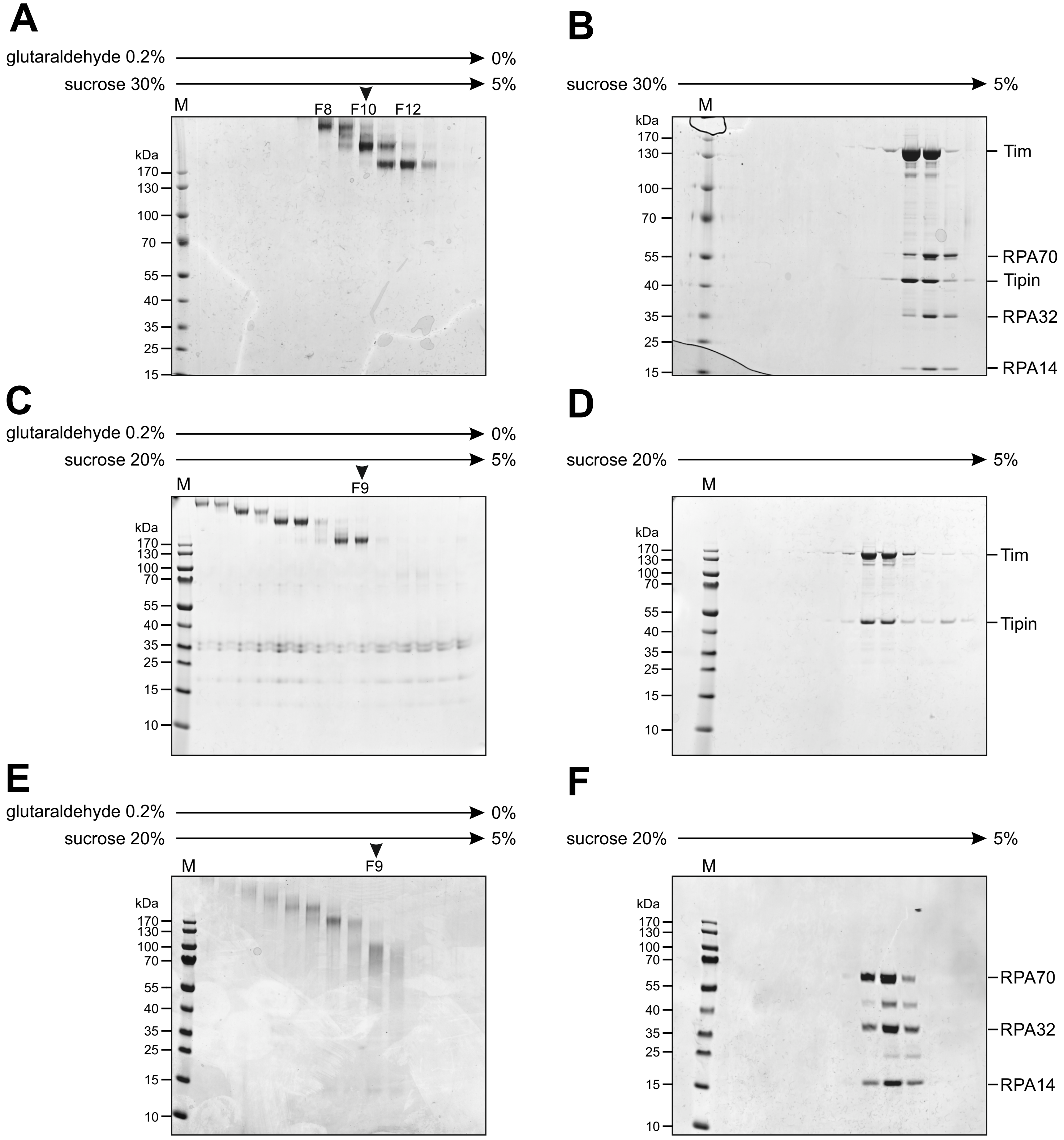

Supplementary Figure 3: SDS-PAGE analysis of the grafix complexes.

Fractions of sucrose/glutaraldehyde gradient centrifugations of (A) cross-linked Tim-Tipin-RPA, (B) non-cross-linked Tim-Tipin-RPA, (C) cross-linked Tim-Tipin, (D) non-cross-linked Tim-Tipin, (E) cross-linked RPA and (F) non-cross-linked RPA were analysed by SDS-PAGE (4-12% Bis-Tris, Invitrogen). Fractions lacking glutaraldehyde were used as control. Fractions indicated by arrows were selected for EM analysis. Tim-Tipin-RPA fraction F8, F10 and F12, Tim-Tipin fraction F9 and RPA fraction F9 were used for mass spectrometry. The stoichiometry of Tim-Tipin-RPA in the selected sample (F10) was calculated to be 1:1 (Tim-Tipin to RPA) using quantitative mass spectrometry analysis (iBAQ) as described in Methods section. (M) Molecular weight marker.

Supplementary Figure 4

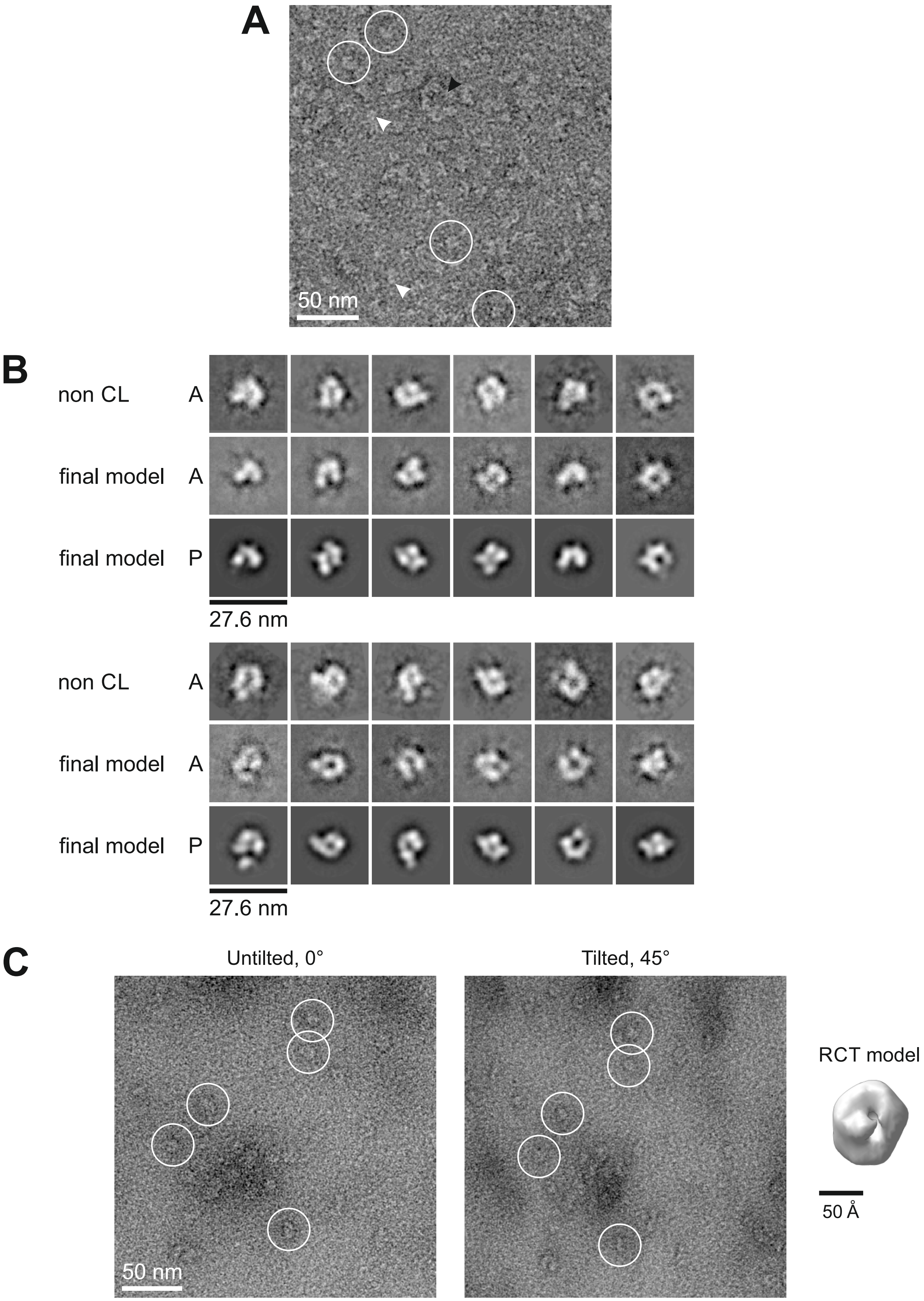

Supplementary Figure 4: Negative stain and Cryo-EM analysis of Tim-Tipin-RPA.

(A) Negative stain image of non-cross-linked Tim-Tipin-RPA. Examples of the complex are marked with white circles. The black and white arrowhead mark an aggregate and partial dissociation of the untreated complex.

(B) Comparison between reference-free 2D class averages (A) of non-cross-linked Tim-Tipin-RPA (non CL), the reference-free 2D class averages of the cryo-EM model (final model, A) and the reprojections of the cryo-EM model (final model, P).

(C) Pair of negative stain images of the same specimen area of cross-linked Tim-Tipin-RPA untilted and tilted to 45°. Examples of the complex are marked with white circles. Initial 3D model obtained using RCT.

Supplementary Figure 5

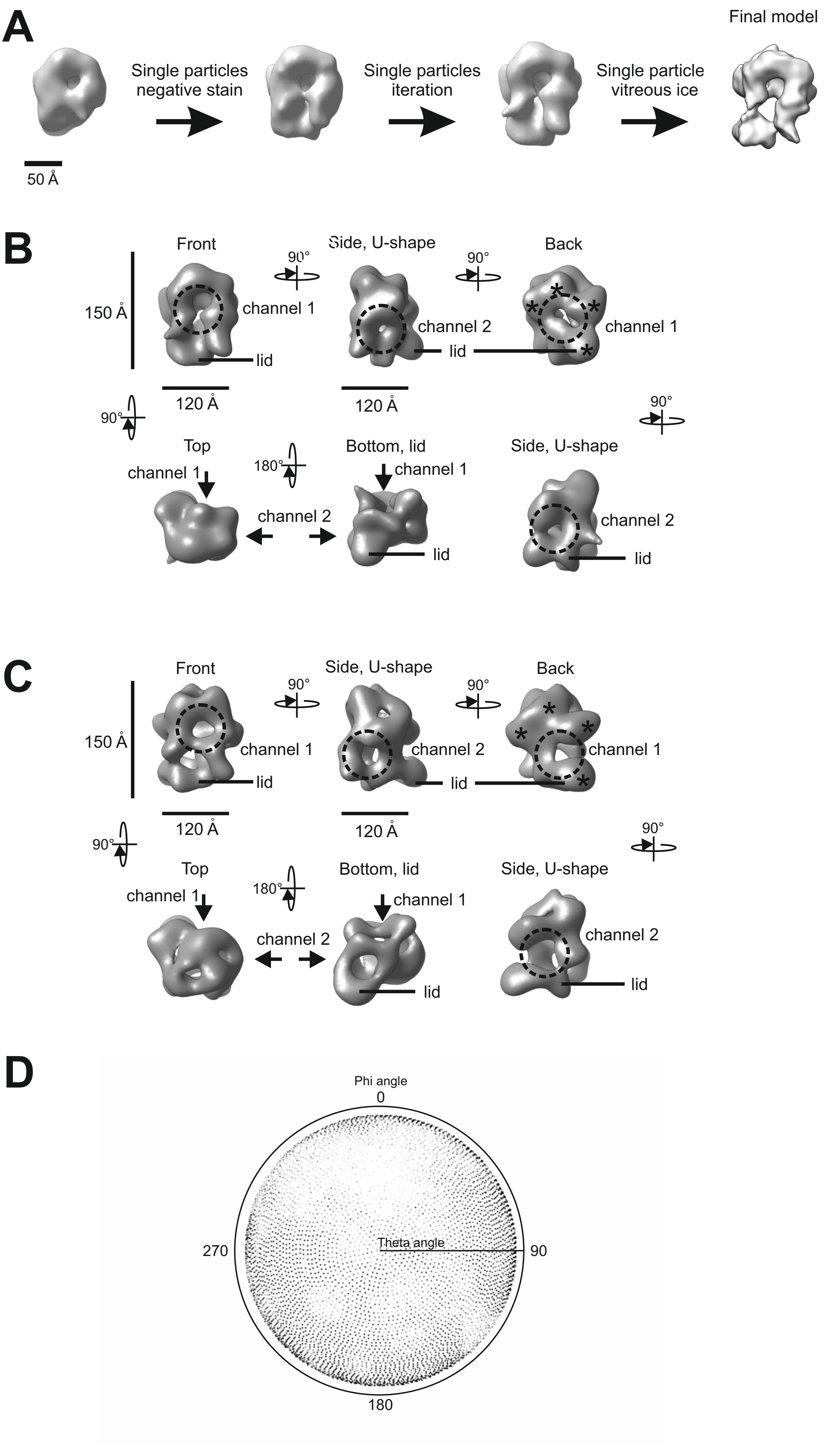

Supplementary Figure 5: 3D reconstructions of Tim-Tipin-RPA.

(A) Refinement of 3D reconstructions. The initial RCT model (Supplementary Figure 4C) was first refined by projection matching using the 2D class averages of 857 untilted particles, then refined using individual particles. After iterative refinement the model was finally refined by projection matching against the cryo-EM raw images of Tim-Tipin-RPA.

(B) Negative stain 3D reconstructions of cross-linked Tim-Tipin-RPA in different views related by rotation around the y- and x-axis as indicated. The major density in the reconstruction displays a ring-like structure (front) containing a ~30-Å-wide channel (channel 1, dashed circle) being closed by a lid density at the bottom (lid). Rotating the described view by 90° unveiled a connection between the major ring-like density and the lid, forming a U-like feature. The protruding density of the ring-like core to the back and the lid form a second channel (channel 2, dashed circle). The view from the back reveals four different domains (marked by stars). The lid volume and the scale bar are marked.

(C) Negative stain 3D reconstructions of the non-cross-linked Tim-Tipin-RPA complex in different views as in (B).

(D) Angular distribution plot of the particles in the final step of refinement. The intensity of the dots is proportional to the number of particles assigned to a certain reference reprojection by projection matching.

Supplementary Figure 6

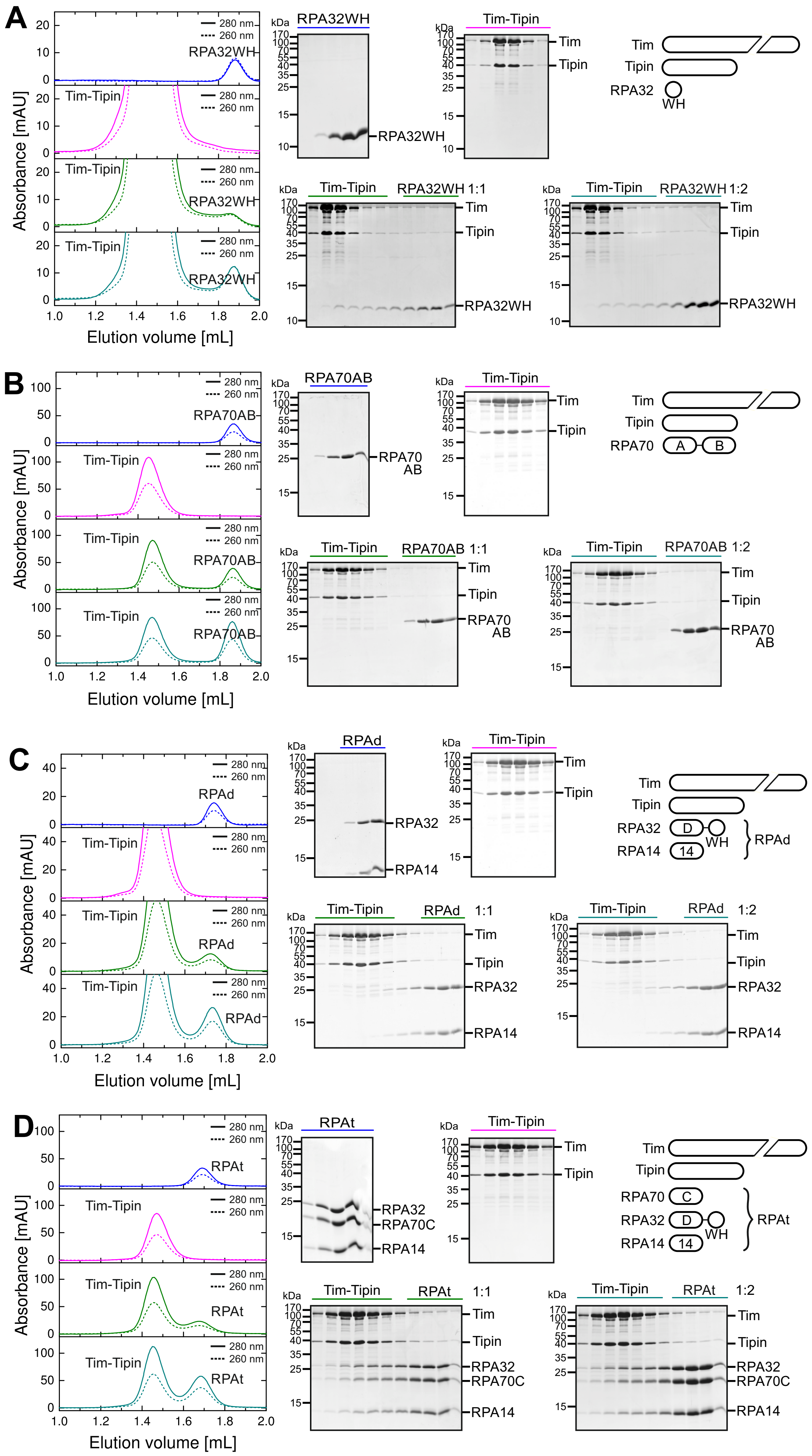

Supplementary Figure 6: Size exclusion chromatography analysis of RPA subunits binding to Tim-Tipin.

Different RPA subunits (blue, (A) RPA32 WH domain, (B) RPA70AB, (C) RPA32/14 (RPAd), (D) RPA70C/32/14 (RPAt)) are mixed with Tim-Tipin at a molar ratio of 1:1 (green) and 2:1 (cyan). No significant complex formation for RPA32WH and RPA32/14 is shown, while RPA70C/32/14 forms a weaker complex compared to RPA70/32/14 (Figure 1B). RPA70AB shows no binding to Tim-Tipin. The elution profile was visualized by UV absorbance at 280 nm (solid) and 260 nm (dashed). The peak fractions for each sample mixture were analysed on Coomassie stained SDS-gels (right). Note that the SEC peak of the WH domain appears smaller, because of low contents of Tyrosine or Tryptophan. For the SDS-PAGE analysis in (A) only one fourth of the sample was used as input for the SDS-gel when compared to (B)-(D). The same SEC run and SDS-gel for Tim-Tipin is illustrated in (B) and (C).

Supplementary Figure 7

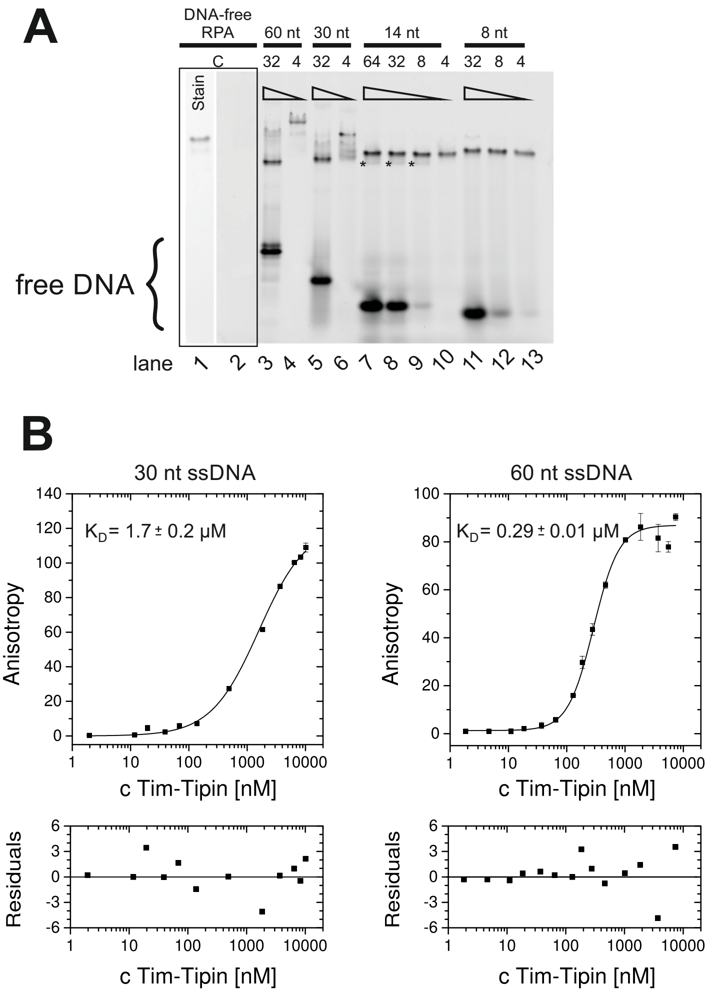

Supplementary Figure 7: Binding of RPA and Tim-Tipin to ssDNA substrates

(A) Electrophoretic mobility shift assay for RPA using a 60 nt, 30 nt, 14 nt and 8 nt 5'-FAM labeled ssDNA substrate. (◣) indicates decreasing amounts of the ssDNA substrate added (total concentration of 64, 32, 8 and 4 μM). The RPA concentration was kept constant at 16 μM. Frame = Control (C). Lane 1 shows the Coomassie stained SDS-gel with DNA-free RPA. Lane 2-13 shows the fluorescence image. The stars in lane 7-9 indicate a small RPA population in the 30 nt binding mode.

(B) Representative binding isotherms for Tim-Tipin binding to 30 nt (left) and 60 nt (right) 5'-FAM labeled ssDNA analysed by fluorescence anisotropy and plotted as a function of protein concentration in log scale. Tim-Tipin is binding with micro-molar (1.7 ± 0.2 μM) to 30 nt and with sub-micro-molar (0.29 ± 0.01 μM) affinity to 60 nt ssDNA. The solid line represents the fit of the binding isotherms using a nonlinear fit to the Hill equation with a Hill coefficient of n = 1.2 ± 0.2 (30 nt) and 2.2 ± 0.1 (60 nt) as described in Materials and Methods. The error bars represent the standard deviation of three independent measurements. (Bottom) Residual plots for the curve fit (difference between the calculated anisotropy of the curve fit and the measured anisotropy) show a good quality of the regressions and no bias.

Supplementary Figure 8

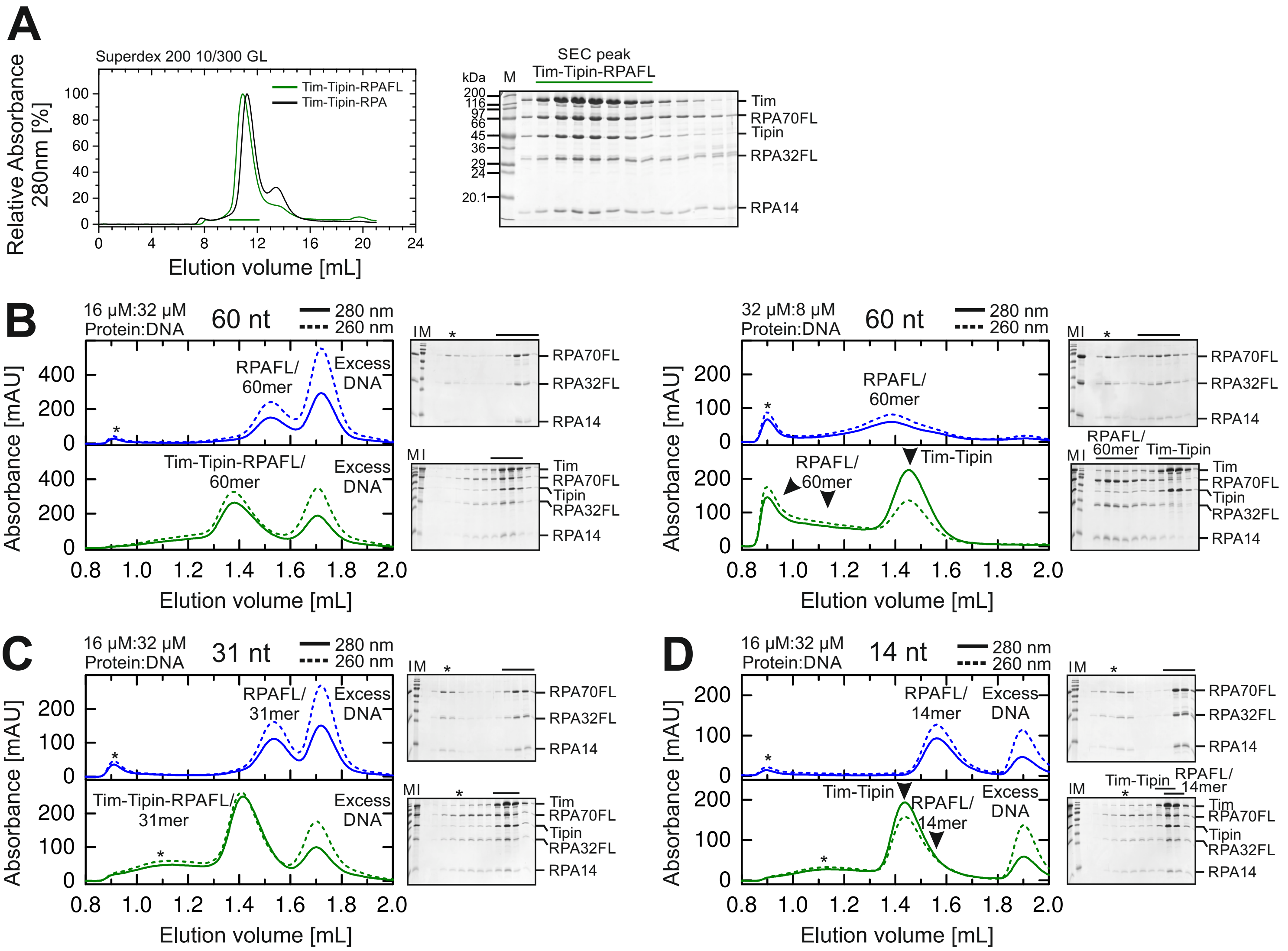

Supplementary Figure 8: Size exclusion chromatography analysis of Tim-Tipin-RPAFL complex formation and binding to ssDNA substrates.

(A) Size exclusion chromatogram (left, green) of reconstituted Tim-Tipin-RPA(full-length) (FL) complex using RPAFL containing the RPA70N and the N-terminal part of RPA32 (RPA32N). For comparison the chromatogram of Tim-Tipin-RPA (lacking the N-terminal regions) has been overlaid (left, black). For complex formation, Tim-Tipin was incubated with a 1.4 molar excess of RPAFL or RPA and loaded on a Superdex 200 10/300 GL column. The elution profile was visualized by UV absorbance at 280 nm and SDS-PAGE. (M) Protein marker in kDa. Note that RPA32FL is partially degraded to a truncated construct lacking the cloning overhang and the first two residues (as shown by N-terminal sequencing).

(B)-(D) Size exclusion chromatograms of RPAFL and Tim-Tipin-RPAFL with (B) 60 nt, (C) 31 nt and (D) 14 nt ssDNA and SDS-gels showing the protein peak fractions (black line). (I) Protein mixture loaded on SEC. (M) Protein marker. Solid line: UV absorbance at 280 nm. Dashed line: UV absorbance at 260 nm. The indicated protein:ssDNA molar ratio have been loaded on a Superose 6 3.2/PC column on ÄKTAmicro. Under SEC conditions we observed a similar behavior of Tim-Tipin-RPAFL compared to Tim-Tipin-RPA lacking the N-terminal RPA70 and RPA32 regions. In particular, under these conditions we see a comparable association/dissociation behavior as a function of DNA length and protein to ssDNA ratio suggesting that the N-terminal regions of RPA70 and RPA32 do not affect Tim-Tipin-RPAFL complex formation and ssDNA binding. The slightly changed 260/280 ratio for Tim-Tipin-RPAFL using the 31 nt ssDNA (260/280 = 1.05) (C) is most likely due to oligomerization of RPAFL (marked by star). Further, we have observed a small population of oligomers of RPAFL eluting at 0.9 mL and 1.0 to 1.2 mL (marked by a star), which was not observed for Tim-Tipin-RPA. This population is further increased, when the RPA-FL concentration is raised to 32 μM (Supplementary Figure 8B, right).

Supplementary Table 1: Intensity based absolute quantification (iBAQ)

	Fraction F8	Fraction F10	Fraction F12
Protein	log Intensity	log Intensity	log Intensity
RPA14	6.67	7.14	6.13
RPA32	6.41	7.09	6.02
RPA70	6.68	7.18	6.19
Tim	7.06	7.35	7.53
Tipin	6.74	7.00	7.19
average			
RPA	6.6 ± 0.2	7.1 ± 0.1	6.1 ± 0.1
Tim-Tipin	6.9 ± 0.2	7.2 ± 0.3	7.4 ± 0.2
